# Regulation of macrophage activity by surface receptors contained within *Borrelia burgdorferi*-enriched phagosomal fractions

**DOI:** 10.1371/journal.ppat.1008163

**Published:** 2019-11-18

**Authors:** Ana Carreras-González, Diego Barriales, Ainhoa Palacios, Marta Montesinos-Robledo, Nicolás Navasa, Mikel Azkargorta, Ainize Peña-Cearra, Julen Tomás-Cortázar, Iraide Escobes, Miguel Angel Pascual-Itoiz, Jana Hradiská, Jan Kopecký, David Gil-Carton, Rafael Prados-Rosales, Leticia Abecia, Estíbaliz Atondo, Itziar Martín, Aize Pellón, Félix Elortza, Héctor Rodríguez, Juan Anguita

**Affiliations:** 1 Inflammation and Macrophage Plasticity Laboratory, CIC bioGUNE, Derio, Bizkaia, Spain; 2 Proteomics Platform, CIBERehd, ProteoRed-ISCIII, CIC bioGUNE, Derio, Bizkaia, Spain; 3 Faculty of Science, University of South Bohemia, Branišovská, České Budějovice, Czech Republic; 4 Structural Biology Unit, CIC bioGUNE, Derio, Bizkaia, Spain; 5 Ikerbasque, Basque Foundation for Science, Bilbao, Bizkaia, Spain; University of Montana, UNITED STATES

## Abstract

Macrophages mediate the elimination of pathogens by phagocytosis resulting in the activation of specific signaling pathways that lead to the production of cytokines, chemokines and other factors. *Borrelia burgdorferi*, the causative agent of Lyme disease, causes a wide variety of pro-inflammatory symptoms. The proinflammatory capacity of macrophages is intimately related to the internalization of the spirochete. However, most receptors mediating this process are largely unknown. We have applied a multiomic approach, including the proteomic analysis of *B*. *burgdorferi*-containing phagosome-enriched fractions, to identify surface receptors that are involved in the phagocytic capacity of macrophages as well as their inflammatory output. Sucrose gradient protein fractions of human monocyte-derived macrophages exposed to *B*. *burgdorferi* contained the phagocytic receptor, CR3/CD14 highlighting the major role played by these proteins in spirochetal phagocytosis. Other proteins identified in these fractions include C-type lectins, scavenger receptors or Siglecs, of which some are directly involved in the interaction with the spirochete. We also identified the Fc gamma receptor pathway, including the binding receptor, CD64, as involved both in the phagocytosis of, and TNF induction in response to *B*. *burgdorferi* in the absence of antibodies. The common gamma chain, FcγR, mediates the phagocytosis of the spirochete, likely through Fc receptors and C-type lectins, in a process that involves Syk activation. Overall, these findings highlight the complex array of receptors involved in the phagocytic response of macrophages to *B*. *burgdorferi*.

## Author summary

Macrophages eliminate infecting microorganisms through the concerted action of surface receptors and signaling molecules. As a consequence, these cells produce a series of soluble factors that participate in the inflammatory response during infections. The composition of the full complement of receptors that participate in the recognition and internalization of the causative agent of Lyme disease, *Borrelia burgdorferi*, is largely unknown. We have analyzed the protein composition of phagosomes containing *B*. *burgdorferi* from human macrophages and identified a series of surface proteins that may be involved in the process. Through the use of gene silencing techniques, we have determined the participation of several of these receptors both in the internalization of the bacterium and the subsequent inflammatory response. Among these, we have identified the Fc gamma receptor pathway as involved in this process in the absence of antibodies. We have also identified receptors that are directly involved in the attachment of *B*. *burgdorferi*, while others seem to have an accessory role in the internalization and/or induction of proinflammatory cytokines in response to the spirochete. These data clarify the complex array of interactions between macrophages and *B*. *burgdorferi* and shed light on the overall response to this infectious agent.

## Introduction

*Borrelia burgdorferi*, the causative agent of Lyme borreliosis, causes a wide variety of pro-inflammatory symptoms. Lyme borreliosis is the most common arthropod-borne infection in Eurasia and North America, with a recent estimate of between 650,000 and 850,000 cases in Europe [1] while around 300,000 cases may be occurring in the United States [2]. Early disease is characterized by flu-like symptoms such as fever, myalgia, and arthralgia. If not correctly diagnosed and treated, the disease evolves into a more complex, multisystemic infection leading to arthritis, carditis, neuroborreliosis, or cutaneous lesions [3–5]. Once disseminated, the infection becomes more refractory to antibiotic treatment [6] while spirochetal elimination by antibodies is less efficient [7, 8].

Innate immune responses, including those by macrophages and neutrophils, not only constitute the first line of defense against the spirochete but also seem to play a critical role once the spirochete has disseminated to distant organs [9]. Phagocytosis ensures the degradation of the pathogen and results in the induction of signaling events leading to the generation of pro-inflammatory cytokines and antigen presentation, which leads to the stimulation of adaptive immunity. In spite of its importance, there are large gaps in our understanding of the receptors involved in the process. While a few surface receptors have been nominally associated with the internalization of the spirochete, such as uPAR [10] or MARCO [11], the only *bona fide* phagocytic receptor described for *B*. *burgdorfei* is composed of the surface proteins Complement Receptor (CR) 3 and CD14 [12–14]. A large proportion of phagocytosis of *B*. *burgdorferi* depends on signals emanating from MyD88. However, the receptors associated with this pathway are currently unknown. MyD88-induced signals are required for the *B*. *burgdorferi*-induced upregulation of MARCO [11]. In contrast, CR3/CD14-mediated phagocytosis is independent of MyD88-induced signals and results in the downmodulation of the proinflammatory responses [14]. In fact, the repression of CR3 surface expression by the TLR family member, CD180, results in the effective modulation of both phagocytosis and proinflammatory responses [15]. Other signaling pathways have been described to be involved in the internalization of *B*. *burgdorferi*, including Syk activation elicited by MARCO [16].

A wide array of surface receptors are involved in the phagocytic internalization of microorganisms, including integrins, C-type lectins, scavenger receptors or Siglecs [17–19]. In the presence of opsonins (complement-derived products and antibodies), phagocytosis can also occur through specific receptors. However, both complement [20] and Fc receptors [21] have been shown to mediate the internalization or cell invasion of pathogens in the absence of opsonins, including enteropathogenic *E*. *coli* [22], a process that requires the high affinity Fc receptor, CD64. For each particular pathogen, its interaction with phagocytic cells is likely to be complex and involve several independent interactions. These, in turn, would likely result in the initiation of several signaling pathways that converge and provide a specific cellular output. In the case of *B*. *burgdorferi*, we have demonstrated that the response of macrophages to the spirochete is dependent on multiple signaling pathways [15] indicating that the array of receptors that trigger these responses is more complex than those already described.

We have identified a group of surface receptors that are involved in both the internalization and the induction of cytokines by *B*. *burgdorferi*. The analysis of the proteome of phagosome-enriched fractions containing the spirochete plus the use of multi-omic data previously generated [15] allowed us to define a set of receptors that modulate the uptake and/or proinflammatory cytokine production of macrophages in response to *B*. *burgdorferi*. The identification of the receptors involved in the response of phagocytic cells to the spirochete reveals the complex interaction of the microorganism and the innate immune system.

## Results

### Isolation and characterization of *B*. *burgdorferi*-containing, phagosome-enriched fractions

**Fig 1:**
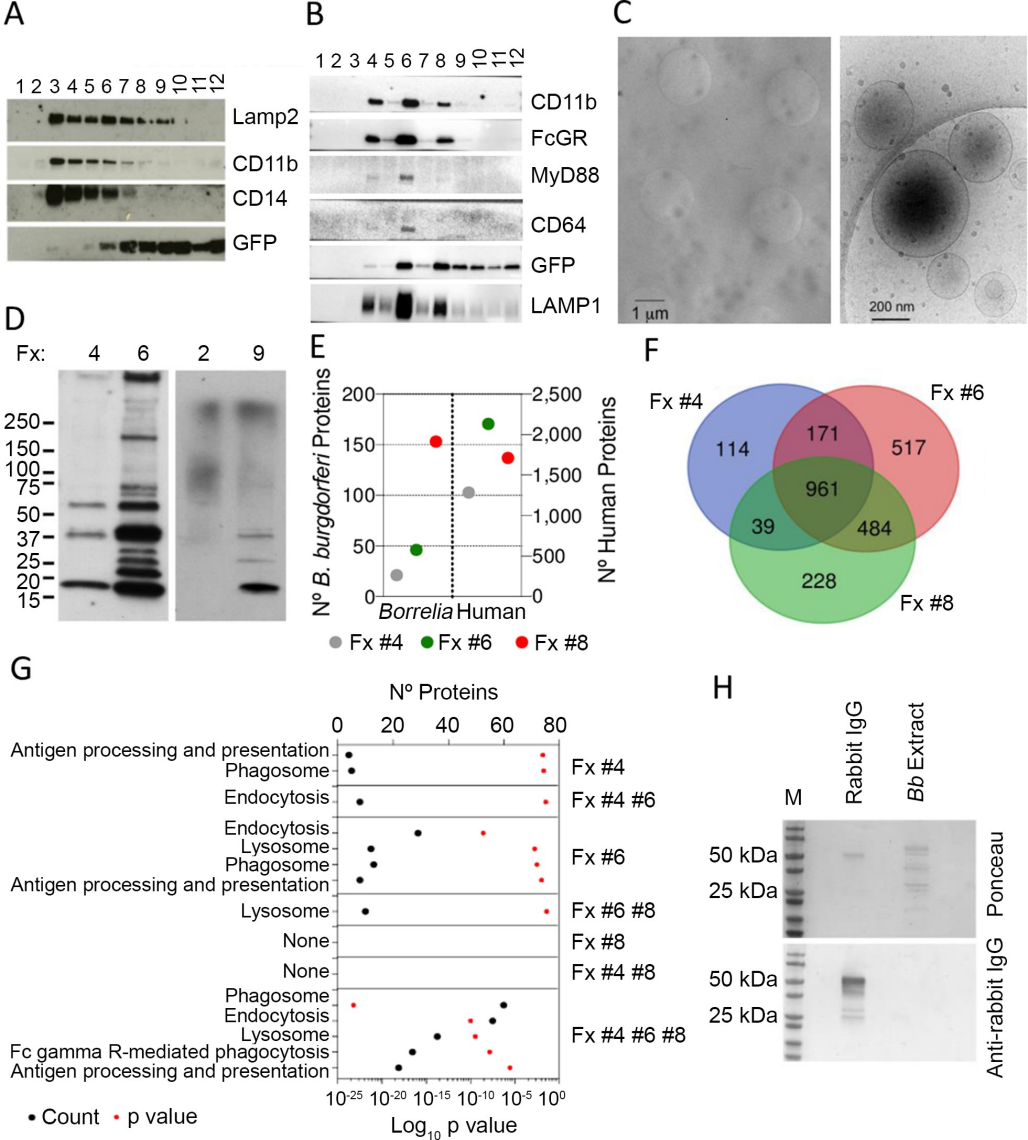
Proteomic characterization of *B*. *burgdorferi*-containing, phagosome-enriched fractions. Immunoblot analysis of *B*. *burgdorferi*-containing phagosome enriched sucrose fractions from murine BMMs (A) and human monocyte-derived macrophages (B). The numbers at the top indicate the number of fraction collected from the top. (C) Cryo-electron micrographs of phagosome-enriched fractions from hMACs. The panel on the left shows an image captured at 4,000 X with phagosomes (dark circles). The clear circles represent the holes of the perforated support foil with pre-defined hole size, shape and arrangement. The image on the right shows plasma membrane-derived liposomes at a higher magnification (30,000 X). (D) Immunoblot using *B*. *burgdorferi*-infected sera of fractions containing GFP (4, 6, 9) or not from (B). (E) Number of *B*. *burgdorferi* and human proteins identified in phagosome-enriched fractions 4, 6 and 8 of hMACs. (F) Venn diagram showing the overlap in protein composition of hMAC phagosome-enriched fractions 4, 6 and 8. A total of 2514 proteins were identified in all 3 fractions, with 961 shared proteins. (G) KEGG pathway analysis of the proteins identified in each fraction. The proteins analyzed correspond to those represented only in each individual fraction, those shared by 2 fractions and the 961 common proteins found in all 3 fractions, as in Fig 1D. (H) Immunoblot of *B*. *burgdorferi* extracts (5 μg) obtained from preparations used in the phagocytosis assays. Rabbit IgG (1 μg) was used as control. The blots were tested with an anti-rabbit IgG antibody. The upper panel shows the staining with Ponceau.

In order to characterize the protein content of cellular fractions enriched in *B*. *burgdorferi*-containing phagosomes, we purified protein extracts of bone marrow-derived macrophages that had been exposed to GFP-containing spirochetes for 2 h by sucrose gradient centrifugation. Immunoblotting analysis showed, as expected [13], the presence of CD11b and CD14 in the lysosome-associated membrane protein 2 (LAMP2)-enriched fractions. These fractions were also positive for GFP (Fig 1A). Similar results were obtained when analyzing human monocyte-derived macrophages (Fig 1B). Further analysis showed the presence of MyD88 in the LAMP1-enriched fractions (Fig 1B). We therefore hypothesized that these fractions would contain *B*. *burgdorferi* phagosomes. Cryo-electron microscopy analysis of phagosome-containing fractions from hMACs showed the presence of phagosomes as structures of around 1 μm, while some plasma membrane-derived liposomes were still present in the preparations (Fig 1C). We determined the proteomic composition of fractions 4, 6 and 8 of the hMac sucrose gradient preparation, because they also contained GFP, indicating the presence of borrelial proteins (Fig 1B and 1D). The identification of several bacterial proteins in these fractions confirmed the presence of phagosomes (Fig 1E). In fact, the number of spirochetal proteins detected correlated with the buoyancy of the *B*. *burgdorferi*-containing fractions (23 proteins in fraction 4, 50 in fraction 6 and 156 in fraction 8, representing normalized spectral abundance factors -NSAF- of 0.7, 2.4 and 6.7, respectively). This suggested a distinct level of degradation of *B*. *burgdorferi* that may be related to the level of maturation of the phagosome/phagolysosome. A total of 2514 proteins were identified in fractions 4, 6 and 8, of which 961 were shared proteins (Fig 1F). Among the identified proteins, we determined the presence of the two components of the phagocytic receptor for *B*. *burgdorferi*, CR3 (ITGAM and ITGB2), as well as CD14, which were present in all fractions (Table 1), confirming a major role for CR3/CD14 in the phagocytosis of *B*. *burgdorferi* [14]. Of note, both TLR2 and TLR8 were present in the three fractions analyzed, as expected [23] (Table 1), while fraction 4 also contained TLR6 and TLR5 (Table 1). The TLR family member CD180, which regulates the phagocytosis of *B*. *burgdorferi* [15] was also present in the three fractions analyzed (Table 1). Analysis of the proteins identified showed the enrichment of components related to phagosome, endocytosis or antigen processing and presentation, particularly in fraction 6 and the pool of common proteins among the three fractions analyzed (Fig 1G). Surprisingly, 27 identified proteins common to the three fractions belonged to the Fc gamma R-mediated phagocytosis pathway (Fig 1G). Since the phagocytosis assays were performed in the absence of serum, and therefore antibodies, these results suggest a role for this pathway in the internalization of opsonin (antibody)-free spirochetes. Furthermore, even though *B*. *burgdorferi* was grown in the presence of rabbit serum, analysis by immunoblotting indicated the lack of attached antibodies molecules in the bacterial preparations used in these assays (Fig 1H).

**Table 1:** Identification of components of phagocytosis and TLRs in phagosome-containing fractions of human monocyte derived macrophages. ND, not detected. PSM: Protein Spectra Matching; SAF: Spectral Abundance Factor; NSAF: Normalized Spectral Abundande Factor.

Uniprot ID	Description	Fraction #	Σ Coverage	Σ# Proteins	Number of Unique Peptides	Number of Total Peptides	Σ# PSMs	SAF	NSAF
ITAM_HUMAN	Integrin alpha-M	4	16.49	1	14	15	41	0.3226	0.1322
		6	23.78	1	21	22	57	0.4485	0.0746
		8	17.71	1	15	16	43	0.3383	0.0707
ITB2_HUMAN	Integrin beta-2	4	37.58	1	24	25	83	0.9796	0.4016
		6	44.47	1	26	27	125	1.4753	0.2455
		8	40.96	1	26	27	96	1.1331	0.2368
CD14_HUMAN	Monocyte differentiation antigen CD14	4	22.40	1	8	8	32	0.7990	0.3275
		6	32.80	1	10	10	67	1.6729	0.2784
		8	32.80	1	10	10	47	1.1735	0.2452
TLR2_HUMAN	Toll-like receptor 2	4	13.65	1	10	10	32	0.3564	0.1461
		6	24.74	1	15	15	58	0.6460	0.1075
		8	14.80	1	10	10	33	0.3676	0.0768
TLR8_HUMAN	Toll-like receptor 8	4	3.94	1	4	4	4	0.0334	0.0137
		6	8.74	1	9	9	14	0.1169	0.0195
		8	7.01	1	7	7	9	0.0752	0.0157
TLR6_HUMAN	Toll-like receptor 6	4	1.63	1	1	1	2	0.0218	0.0089
		6	ND	ND	ND	ND	ND	ND	ND
		8	ND	ND	ND	ND	ND	ND	ND
TLR5_HUMAN	Toll-like receptor 5	4	0.93	1	1	1	1	0.0102	0.0042
		6	ND	ND	ND	ND	ND	ND	ND
		8	ND	ND	ND	ND	ND	ND	ND
CD180_HUMAN	CD180 Antigen	4	2.42	1	1	1	1	0.0135	0.0055
		6	8.47	1	4	4	7	0.0944	0.0157
		8	2.42	1	1	1	2	0.0270	0.0056

### The analysis of the *B*. *burgdorferi*-induced transcriptome reveals the regulation of several putative phagocytic receptors

**Fig 2:**
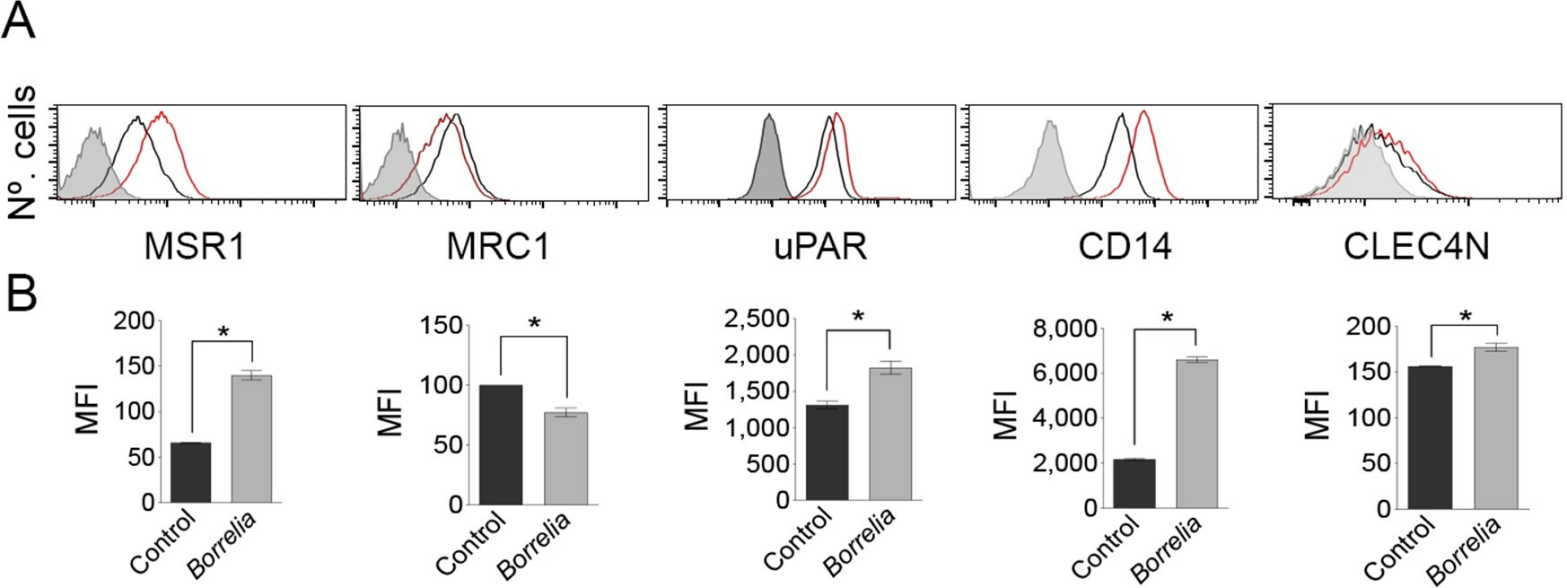
Validation of the transcriptomic analysis of BMM receptors involved in the response to *B*. *burgdorferi*. (A) Flow cytometry analysis of receptors after 16 h stimulation with *B*. *burgdorferi* (red histogram / grey bar) or non-treated controls (black histogram / black bar). The gray histogram represents the 4°C control. (B) Histograms showing the mean fluorescence intensity (MFI) of BMM stimulated with *B*. *burgdorferi* and controls. The data shown represent the average ± SE of BMM from 3 mice.

We have recently described the regulation of macrophage surface receptors in response to the stimulation with *B*. *burgdorferi* that modulate both the capacity of these cells to phagocytose the spirochete and the ensuing inflammatory response [15]. *B*. *burgdorferi* induces the downregulation of CD180, leading to increased expression of CR3, augmented phagocytosis and reduced proinflammatory cytokine production [15]. On the other hand, the C-type lectin receptor, Mincle, did not influence either phagocytosis of, or TNF production in response to *B*. *burgdorferi*. In order to identify additional surface receptors that modulate either aspect of the macrophage response, we further analyzed the multi-omic murine and human data previously reported [15]. Table 2 shows the comparative transcriptomic analysis of several members of different classes of receptors that may be involved in the response of monocytes/macrophages to *B*. *burgdorferi*. We analyzed the regulation of C-type lectins, GPI anchored proteins, Scavenger receptors, Siglecs and Fc receptors. Several receptors belonging to these families where regulated by the stimulation with *B*. *burgdorferi* (Table 2). The validation by flow cytometry of the surface upregulation of these receptors is presented in Fig 2. Importantly, several of these receptors were found in the phagosome-enriched fractions (Table 2), further suggesting their potential role in the interaction of macrophages and the spirochete.

**Table 2:** Regulation of surface receptors by stimulation of BMMs and hMon with *B*. *burgdorferi*, as reported [15]. The transcriptomic and microarray data correspond to GEO accession number GSE103483. The proteomics data of BMM correspond to the ProteomeXchange accession number PXD008228. The last column indicates the identification of this receptors in phagosome-enriched fractions of hMACs stimulated with the spirochete. WB: immunoblotting, NP, not present.

RNAseq BMM	Fold Change	p Value	Proteomics BMM	Fold Change	Anova (p)	μARRAY hMon	Fold Change	p Value	Phagosome
FcgR associated									
*Fcer1g*	1.03	8.40E-01	FCERG	0.91	8.39E-01	*FCER1G*	2.15	1.23E-04	YES
*Fcgr1*	1.25	4.92E-01	FCGR1A	0.62	3.79E-02	*FCGR1A*	0.60	1.15E-03	YES (WB)
NP			FCGR2	4.63	7.22E-03	*FCGR2A*	3.72	7.15E-05	YES
*Fcgr2b*	6.54	2.48E-23	NP			*FCGR2B*	1.54	1.19E-01	YES
*Ptprc*	0,82	8.54E-02	PTPRC	0.73	2.23E-01	*PTPRC*	0.83	8.94E-03	YES
*Syk*	1.49	6.54E-24	KSYK	1.18	2.61E-01	*SYK*	0.87	1.06E-01	YES
*Inpp5d*	0.61	1.03E-08	SHIP1	0.74	2.81E-01	*INPP5D*	0.38	3.49E-06	YES
C-type lectins									
*Clec1a*	0.09	1.26E-03	NP			*CLEC1A*	0.84	1.72E-02	NO
*Clec4a3*	0.50	1.89E-03	NP			*CLEC4A*	1.90	2.27E-04	YES
*Clec4b1*	2.95	5.05E-06	NP			NP			NO
*Clec4d*	3.79	8.21E-09	NP			*CLEC4D*	1.01	9.64E-01	NO
*Clec4e*	35.67	9.73E-08	CLC4E	7.50	6.38E-04	*CLEC4E*	2.26	2,.24E-05	NO
*Clec4n*	27.08	7.15E-33	NP			*CLEC4N*	1.02	8.01E-01	NO
*Clec5a*	1.95	9.87E-03	NP			*CLEC5A*	2.80	6.97E-06	YES
*Clec7a*	0.15	1.84E-38	NP			*CLEC7A*	0.71	4.31E-04	YES
*Clec10a*	0.34	2.29E-03	NP			*CLEC10A*	0.57	3.84E-03	NO
*Clec12a*	0.11	3.46E-155	NP			*CLEC12A*	2.50	6.01E-03	NO
*Cd302 (Clec13a)*	4.47	1.36E-08	NP			*CD302*	0.26	1.36E-04	YES
*Ly75 (Clec13b)*	7.73	9.11E-06	NP			NP			YES
*Mrc1*	0.11	4.95E-27	MRC1	0.77	5.33E-01				YES
Scavenger receptors									
*Stab1*	0.45	7.75E-09	NP			*STAB1*	0.27	1.56E+05	YES
*Stab2*	0.13	2.35E-04	NP			NP			NO
*Marco*	2.00	4.87E-01	NP			*MARCO*	0.49	4.09E-03	YES
*Msr1*	5.42	2.52E-61	NP			NP			YES
Siglecs									
*Siglec1*	1.31	3.80E-01	NP			*SIGLEC1*	0.89	1.80E-01	YES
*Siglec5*	0.04	1.57E-21	NP			*SIGLEC5*	1.26	3.19E-01	YES
*Cd33*	2.27	3.08E-04	NP			*CD33*	0.23	4.45E-06	YES
GPI Anchored proteins									
*Cd52*	0.62	3.23E-02	NP			*CD52*	0.06	5.33E-07	NO
*Ly6e*	2.57	5.59E-05	NP			NP			NO
*Cd59a*	0.34	3.67E-10	NP			*CD59*	1.74	1.43E-04	YES
*Cd24a*	4.74	1.19E-03	NP			NP			NO
*Cd14*	4.99	3.10E-22	CD14	2.37	6.25E-03	*CD14*	0.85	7.25E-01	YES
*Plaur*	4.96	1.28E-47	NP			*PLAUR*	2.51	5.17E-05	YES
Integrins									
*Itgam*	0.80	3.50E-01	ITGAM	1.17	4.27E-01	*ITGAM*	0.11	3.95E-07	YES
*Itgb2*	1,56	6,69E-06	ITGB2	0,63	2,66E-02	*ITGB2*	0,17	7,19E-07	YES

### Silencing of the potential receptors in RAW 264.7 shows their implication in *Borrelia burgdorferi* phagocytosis

**Fig 3:**
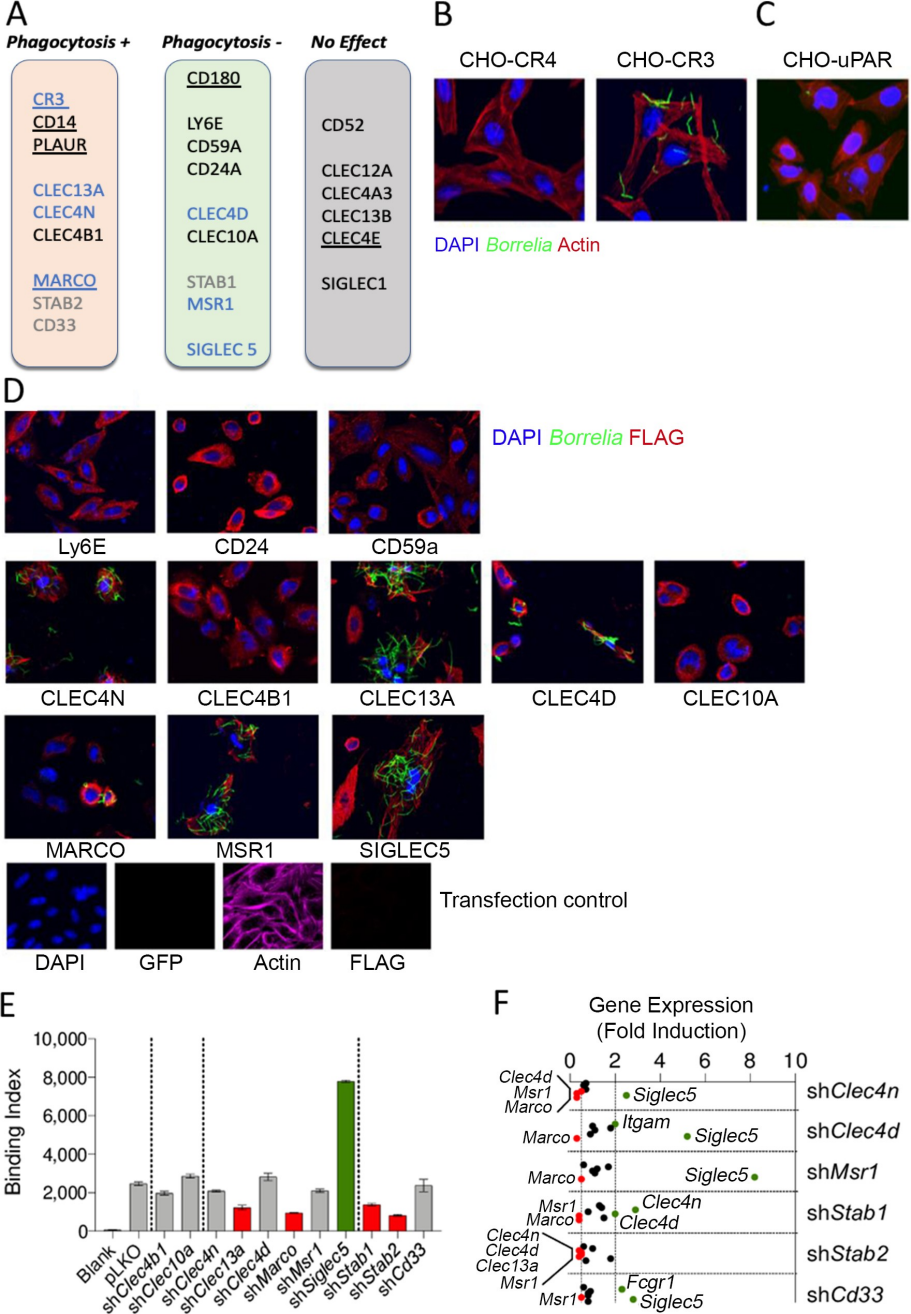
Several surface receptors are involved in the phagocytosis of RAW 264.7 cells to *B*. *burgdorferi*. (A) Schematic representation of the role of the receptors involved positively or negatively in *B*. *burgdorferi* phagocytosis. Confocal micrographs of CHO cells stably transfected with Complement Receptor (CR) 4 and CR3, or transiently transfected with several putative phagocytic receptors (C,D). The cells were incubated in the presence of GFP-*B*. *burgdorferi* (m.o.i. = 100) and stained for nuclei (DAPI), actin (Phalloidin) or the FLAG tag present in the receptors used in D. The bottom panels in D represent a transfection control incubated and stained under the same conditions. (E) *B*. *burgdorferi* binding at 4°C to RAW 264.7 cells expressing shRNA specific for the putative phagocytic receptors. Significantly different binding is represented by red (lower binding) or green (higher binding) bars. The data corresponds to the average ± SE of 3 determinations and are representative of at least 2 independent experiments. (F) Gene expression levels of binding receptors shown to bind B. burgdorferi in panel D that did not show differential binding at 4°C in panel E, as well as those not tested by confocal microscopy (STABILIN1, STABILIN2 and CD33). Receptors that showed expression levels ≤ 0.5 or ≥ 2 relative to pLKO control cells are represented in red and green, respectively.

We next evaluated the role of several of the receptors identified in the response to *B*. *burgdorferi* by overexpression of specific shRNAs in RAW 264.7 cells (S1 Table, S1 Fig). Stable cell lines established after selection of lentivirally infected RAW 264.7 cells were first analyzed for their phagocytic activity compared to vector-transduced controls. The results are presented in S2–S5 Figs and summarized in Fig 3A. Of the receptors analyzed, several (CD52, CLEC12A, CLEC4A3, CLEC13B, and SIGLEC1) did not affect the phagocytosis of *B*. *burgdorferi* (Fig 3A, S2–S5 Figs). Silencing of PLAUR (uPAR), CLEC13A, CLEC4N (Dectin 2), CLEC4B1, MARCO, STABILIN2 or CD33 however, caused a diminished uptake of *B*. *burgdorferi* by RAW 264.7 cells to different extents (45%, 43%, 30%, 45%, 29%, 32%, 22%, respectively; Fig 3A, S2–S5 Figs). These included receptors previously shown to be involved in the internalization of the spirochete, such as MARCO [11] and uPAR [10]. On the other hand, silencing of LY6E (41%), CD59A (197%), CD24A (35%), CLEC4D (37%), CLEC10A (38%), STABILIN1 (181%), MSR1 (37%) or SIGLEC5 (42%) resulted in the increased ability of the cells to internalize the spirochete (Fig 3A, S3–S6 Figs).

We then assessed the capacity of these receptors to directly bind *B*. *burgdorferi*. We expressed ectopically in CHO cells several receptors shown to affect the phagocytosis of the spirochete in silenced RAW 264.7 cells (Fig 3A). As reported [14], CR3 expression resulted in spirochetal binding, while CR4 did not affect microbial attachment to the cells (Fig 3B). The expression of the GPI-anchored proteins, uPAR (Fig 3C), Ly6E, CD24a or CD59a (Fig 3D) did not result in *B*. *burgdorferi* binding. These results suggest that the effect of these proteins, as shown for CD14 [14] is accessory to binding receptors. On the other hand, the C-type lectins CLEC4N, CLEC13A, CLEC4D, the scavenger receptors MARCO and MSR1, and SIGLEC5 were readily able to bind the spirochete when expressed in CHO cells (Fig 3D), while CLEC4B1 or CLEC10A expression did not affect binding (Fig 3D). We also analyzed the ability of silenced RAW 264.7 cells to bind *B*. *burgdorferi* at 4°C. In correlation with the lack of binding in CHO cells, silenced RAW 264.7 cells for *Clec4b1*, *Clec10a* failed to show any differential attachment compared to control silenced cells (Fig 3E). On the other hand, silenced RAW 264.7 cells for *Clec13a* (51%) and *Marco* (63%) bound the spirochete at significantly lower levels than the control cells (Fig 3E). However, cells silenced for *Clec4n*, *Clec4d* and *Msr1* did not show any differential binding activity in spite of being able to bind *B*. *burgdorferi* (Fig 3E). Finally, binding of *B*. *burgdorferi* at 4°C was significantly lower in RAW 264.7 cells silenced for both *Stab1* (46%) and *Stab2* (68%), while *Cd33* did not show an effect in the ability of the cells to bind the spirochete, and *Siglec5* silencing induced a marked increased (208%) in the capacity of the cells to bind the bacterium (Fig 3E).

In order to assess whether gene silencing had affected the expression of the binding receptors (Fig 3D), we analyzed by qRT-PCR the level of expression of *Itgam*, *Fcgr1*, *Clec4n*, *Clec13a*, *Clec4d*, *Marco*, *Msr1* and *Siglec5*. sh*Clec4n*, sh*Clec4d* and sh*Msr1* cells showed increased expression of *Siglec5* (2.5, 5.2 and 8.2 fold, respectively), while the expression levels of other binding receptors, such as *Marco* were reduced in these cells (0.3, 0.3 and 0.5 fold, respectively; Fig 3F). Furthermore, sh*Clec4d* cells showed increased expression of *Itgam* (2 fold), while sh*Clec4n* cells contained reduced expression levels of *Clec4d* (0.5 fold), *Marco* (0.3 fold) and *Msr1* (0.3 fold; Fig 3F). These results suggested that silencing of *Clec4n*, *Clec4d* and *Msr1* had resulted in the differential expression of several binding receptors, with an overall neutral effect on spirochetal binding compared to control silenced cells. The analysis of sh*Stab1* and sh*Cd33* cells showed similar differential expression of several binding receptors (Fig 3F). However, sh*Stab2* cells showed significantly reduced levels of *Clec4n* (0.4 fold), *Clec4d* (0.5 fold), *Clkec13a* (0.5 fold) and *Msr1* (0.4 fold), while no receptor was significantly increased (Fig 3F).

### Distinct effect of surface receptors in TNF production in response to *Borrelia burgdorferi* in silenced RAW 264.7 cells

**Fig 4:**
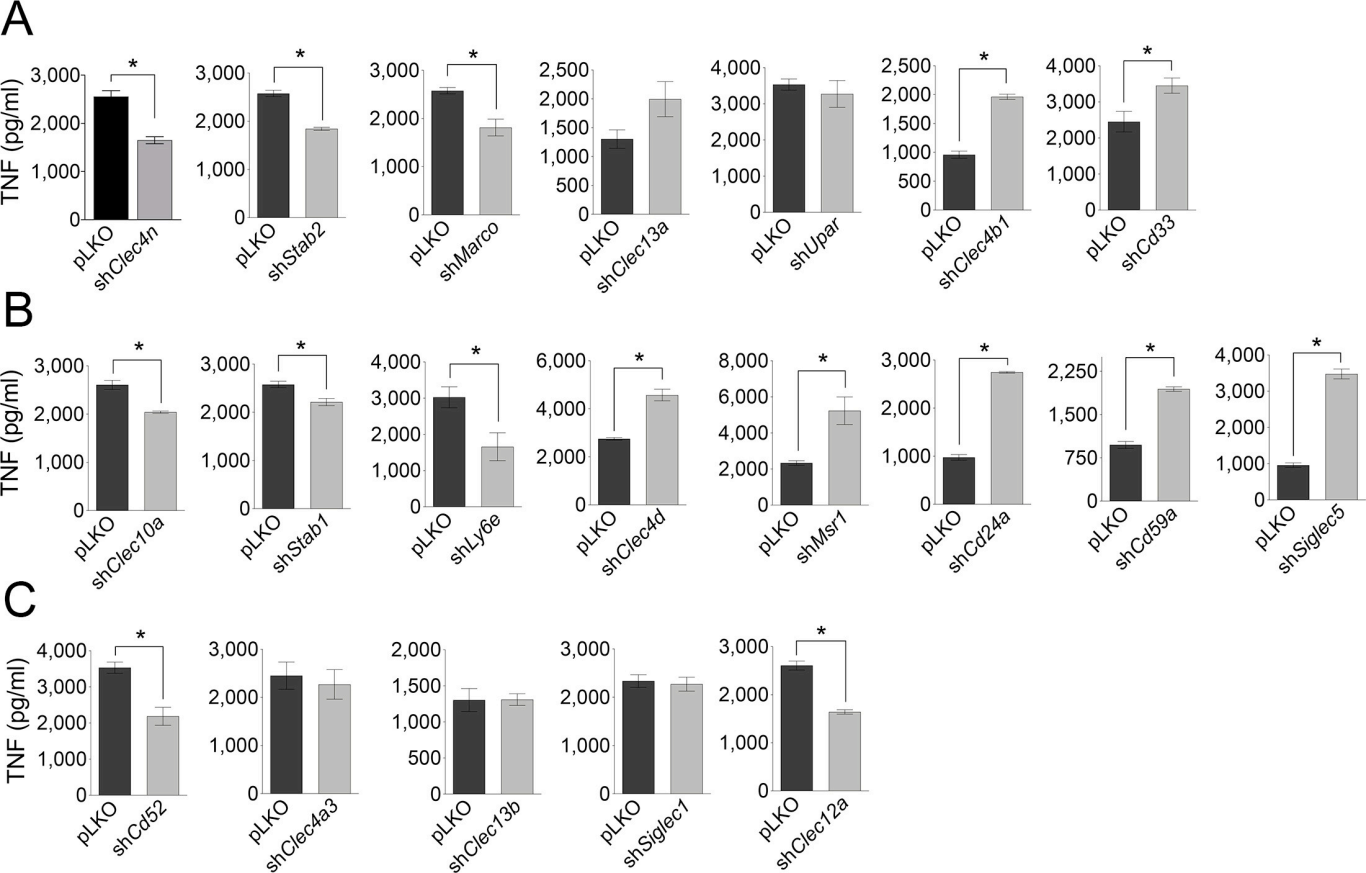
Effect of gene silencing of surface receptors in the induction of TNF in response to *B*. *burgdorferi*. TNF induction by RAW 264.7 cells silenced for the receptors involved positively (A), negatively (B) or not involved (C) in *B*. *burgdorferi* phagocytosis upon stimulation with the bacteria. The data corresponds to the average ± SE of 3 determinations and are representative of at least 3 independent experiments.

We next analyzed the effect of shRNA overexpression on TNF induction in response to *B*. *burgdorferi*. Of the receptors positively associated with *B*. *burgdorferi* phagocytosis, CLEC4N, STABILIN2 and MARCO were also involved in the positive regulation of TNF (35%, 29% and 30%, respectively; Fig 4A), while CLEC4B1 and CD33 negatively regulated the induction of the cytokine (105% and 41%, respectively; Fig 4A). Interestingly, both CLEC13A and uPAR silencing had no effect on the induction of TNF, in spite of their positive regulation of phagocytosis (Fig 4A). Of the receptors negatively regulating the uptake of *B*. *burgdorferi*, CLEC10A, STABILIN1 and LY6E positively regulated the induction of TNF (22%, 14% and 45%, respectively; Fig 4B), while silencing of CLEC4D, MSR1, CD24A, CD59A and SIGLEC5 resulted in increased TNF levels (66%, 124%, 182%, 99% and 263%, respectively; Fig 4B). Of note, although CLEC12A and CD52 silencing did not affect the phagocytic capacity of RAW 264.7 cells, it affected TNF production in response to the spirochete (Fig 4C).

### CD64 mediates the phagocytosis of *B*. *burgdorferi* in absence of specific antibodies

**Fig 5:**
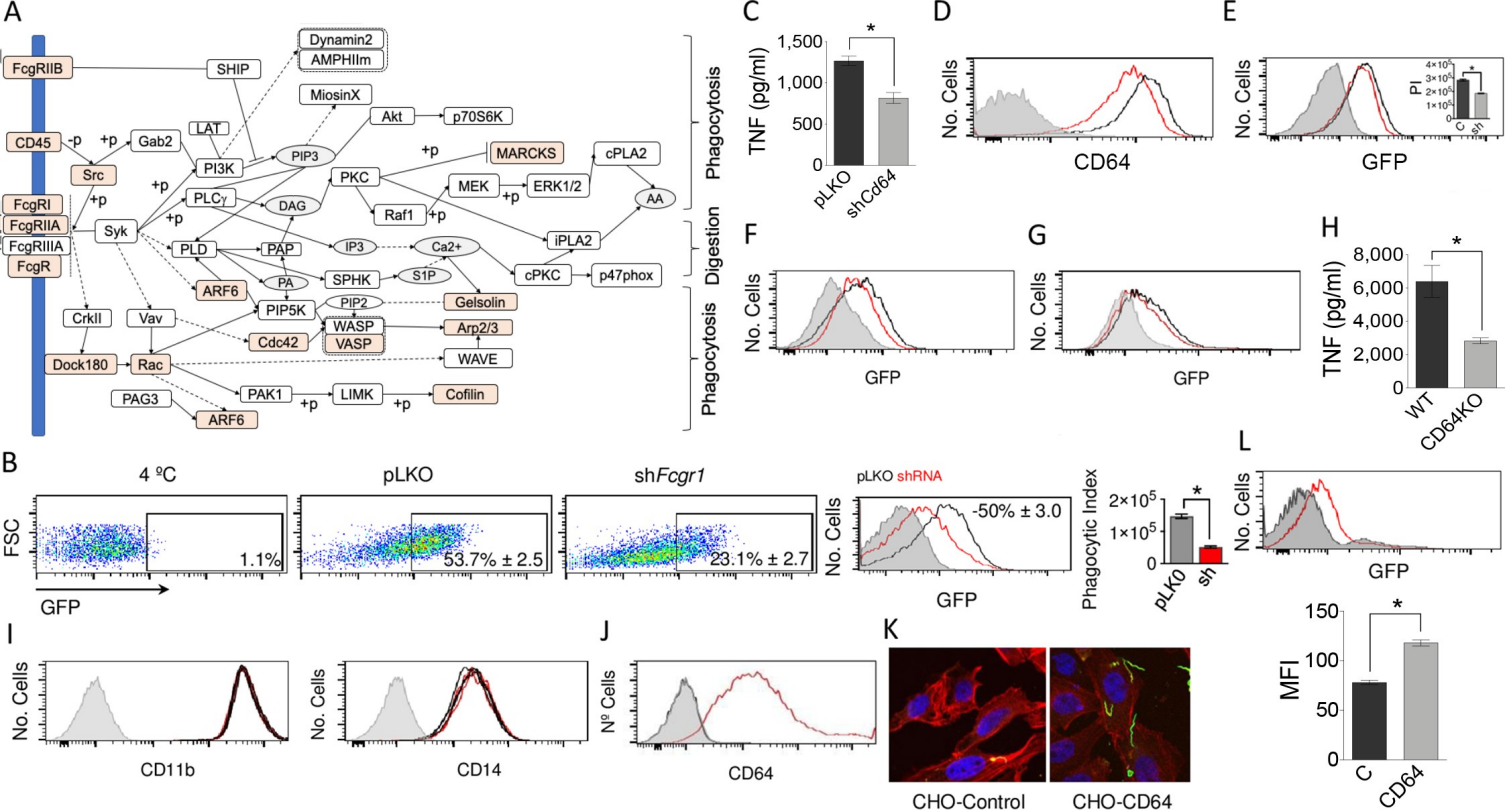
The Fc gamma Receptor phagocytosis pathway is implicated in the response of macrophages to *B*. *burgdorferi*. (A) Components of the KEGG Fc gamma Receptor pathway identified (orange) in the proteomics analysis of the phagosome-enriched fractions of hMAC. (B) (Left panels) Percentage of GFP-positive RAW 264.7 cells in sh*Fcgr1*- (right dot plot) or pLKO.1-infected (middle dot plot) cells. The 4°C control is represented on the left dot plot. (Middle panel) Histograms representing phagocytosis by sh*Fcgr1*- (red histogram) and control-infected (black histograms) RAW 264.7 cells. The gray histogram represents the 4°C control. (right panel) Phagocytic index of sh*Fcgr1*- (grey bar) and pLKO.1-infected (red bar) RAW 264.7 cells. The data represent the average ± SE of 3 determinations. (C) TNF production by sh*Fcgr1a* RAW 264.7 cells (grey bar) and pLKO.1 controls (black bar) upon stimulation with *B*. *burgdorferi*. The average ± SE of 3 determinations is represented. (D) Lower expression levels of CD64 on *Fcgr1a*-silenced BMMs (red histogram) compared to pLKO.1 lentivirally infected cells (black histogram), as determined by flow cytometry. The gray histogram represents the unstained control. (E) Phagocytosis of *B*. *burgdorferi* by sh*Fcgr1a* BMMs (red histogram) or pLKO.1-infected controls (black histogram). The gray histogram represents the 4°C control. The insert shows the average phagocytic index (PI) of 3 independent mice. (F) Phagocytosis of *B*. *burgdorferi* by hMACs in the presence of a blocking antibody targeting CD64 (red histogram) or controls (black histogram). The gray histogram represents the 4°C control. The results are representative of 8 individual donors. (G) Phagocytosis of *B*. *burgdorferi* by BMMs differentiated from CD64-deficient mice (red histogram) or wildtype controls (black histogram). The gray histogram represents the 4°C control. The results are representative of at least 3 independent experiments. (H) TNF production upon stimulation of BMM from CD64-deficient mice (grey bar) or wildtype controls (black bar) with *B*. *burgdorferi*. The data represent the average ± SE of 3 mice per group. (I) Flow cytometry analysis showing the expression of CD11b and CD14 in BMMs from CD64-deficient mice (red histogram) or wildtype controls (black histogram). The gray histogram represents the unstained control. (J) Histogram representing the ectopic expression of human CD64 in CHO cells (red histogram) and un-transfected controls (black histogram). The gray histogram represents the unstained control. (K) Confocal micrograph of un-transfected CHO cells (CHO-Control) and CHO cells transfected with human CD64 (left). The cells were stained with phalloidin (red) and DAPI (blue). *B*. *burgdorferi* are shown in green. (L) Binding of *B*. *burgdorferi* to CHO cells ectopically expressing human CD64 (red histogram) compared to un-transfected cells (black histogram). The gray histogram represents the 4°C control. The average mean fluorescence intensity (MFI) ± SE of 3 determinations is presented on the graph below, and is representative of at least 3 independent experiments.

The proteomics analysis of the phagosome-enriched fractions of hMACs showed a significant contribution of components of the Fcγ phagocytosis pathway (Fig 5A), including CD64 (Table 2). Because Fcγ Receptor I Alpha (FcgRI, CD64) has been shown to directly recognize *Escherichia coli* by murine macrophages in the context of meningeal infection [22] and to mediate bacterial invasion, we further assessed whether this receptor would mediate the internalization of *B*. *burgdorferi* in the absence of opsonins (antibodies). Silencing *Fcgr1* in RAW 264.7 cells resulted in decreased phagocytosis of *B*. *burgdorferi* (Fig 5B) and production of TNF (36%; Fig 5C). *Cd64*-silenced BMMs (Fig 5D) also showed reduced uptake of the spirochete in serum-free medium (35%; Fig 5D). Furthermore, the use of a blocking antibody targeting CD64 resulted in the reduced (63%) phagocytic capacity of human blood monocyte-derived macrophages (Fig 5F). In addition, BMMs from CD64-deficient mice showed a diminished (26%) phagocytic capacity (Fig 5G) and TNF production (56%) in response to *B*. *burgdorferi* (Fig 5H). The attenuated capacity of silenced cells to internalize *B*. *burgdorferi* was not associated with variations in the expression of CD11b or CD14 (Fig 5I). In order to demonstrate the capacity of CD64 to bind the spirochete, we expressed ectopically the receptor in CHO cells (Fig 5J). Compared to non-transfected controls, CHO-CD64 cells were able to bind *B*. *burgdorferi*, demonstrating the capacity of this receptor to anchor the spirochete (Fig 3K and 3L).

### The common gamma chain mediates the phagocytosis of *B*. *burgdorferi*

**Fig 6:**
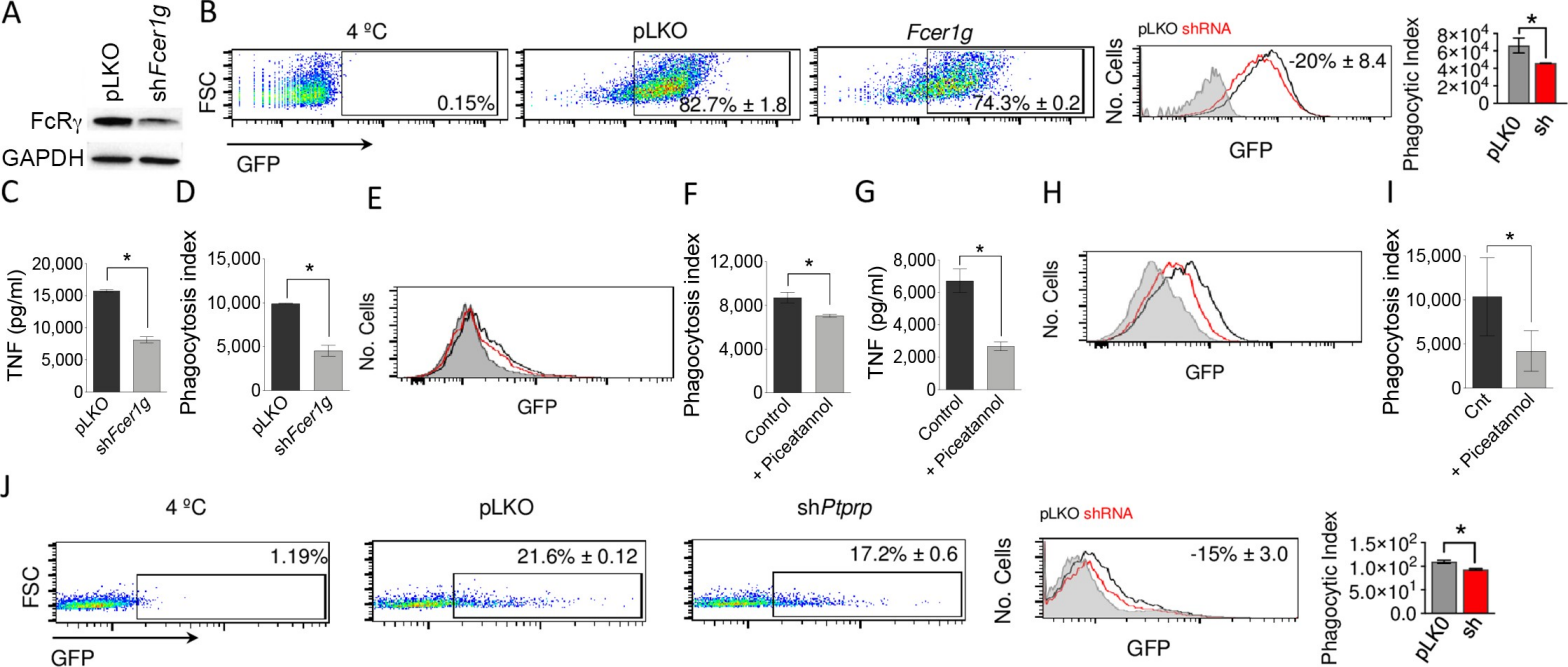
The common gamma chain, Syk kinase and SHIP1 phosphatase are mediators of *B*. *burgdorferi* phagocytosis. (A) Downregulation of FcγR expression in RAW 264.7 cells infected with lentivirus containing sh*Fcer1g* or the empty vector, as determined by immunoblot analysis. (B) Phagocytosis of *B*. *burgdorferi* by sh*Fcer1g* RAW 264.7 cells. (C) TNF production by sh*Fcer1g* RAW264.7 (grey bar) or pLKO.1 controls (black bar) upon stimulation with *B*. *burgdorferi*. The data represent the average ± SE of 3 experiments. (D) Phagocytosis of *B*. *burgdorferi* by sh*Fcerig* BMMs (grey bar) or controls (pLKO.1 infected, black), analyzed as in Fig 5B. The data represent the average ± SE of 3 mice. (E) Phagocytosis of *B*. *burgdorferi* by BMMs treated with Piceatannol (red histogram) or non-treated controls (black histogram). The gray histogram represents the 4°C control. (F) Phagocytosis index of BMMs treated with piceatannol (grey bar) or non-treated controls (black bar). The data represent the average ± SE of 3 mice. (G) TNF production of piceatannol treated BMMs in response to *B*. *burgdorferi* stimulation (grey bar) and controls (black bar). The average ± SE of 3 mice per group is presented. (H) Phagocytosis of *B*. *burgdorferi* by hMACs treated with Piceatannol (red histogram) and controls (black histogram). The gray histogram represents the 4°C control. (I) Phagocytosis index of hMACs treated with Piceatannol (grey bar) and controls (black bar). The data represent the average ± SE of 5 donors. (J) Phagocytosis of *B*. *burgdorferi* by sh*Ptprc* RAW 264.7 cells. The data were analyzed as in Fig 5B and represent the average ± SE of 3 experiments.

Fc and other receptors, including some C-type lectins require the recruitment and signals elicited by the common gamma chain (FCER1G, FcγR) [19, 21, 24]. These include some of those that modulate the internalization of the spirochete, such as CLEC4N, or CLEC4D. We therefore assessed the role of FCER1G in the internalization of the spirochete. RAW 264.7 cells harboring sh*Fcer1g* (Fig 6A) showed a diminished (30%) capacity to internalize *B*. *burgdorferi* (Fig 6B) and resulted in decreased (48%) induction of TNF in response to the spirochete (Fig 6C). The same reduction in phagocytosis to *B*. *burgdorferi* was observed in silenced BMMs (54%; Fig 6D).

The common FcR γ-chain mediates Syk recruitment, which is responsible for downstream signaling activation. Since Syk silencing resulted in the loss of viability of RAW 264.7 cells, we proceeded to the chemical inhibition of the kinase with Piceatannol [16]. Both phagocytosis of *B*. *burgdorferi* (19%; Fig 6E and 6F) and TNF induction (60%; Fig 4G) were decreased in BMMs, confirming the implication of this enzyme in this process. Furthermore, Syk inhibition in hMACs decreased the capacity of these cells to phagocyte *B*.*burgdorferi* (74%; Fig 4H and 4I).

FcgR phosphorylation and Syk recruitment requires the participation of CD45 (PTPRC), through the initial dephosphorylation of Src kinases. Since CD45 was present in the phagosome-enriched fractions of hMACs (Table 2), we also analyzed the involvement of this phosphatase on the internalization of *B*. *burgdorferi*. As expected, *Ptprc* silencing in RAW 264.7 cells resulted in diminished internalization of the spirochete (15%; Fig 6J). Overall, these data show that the Fc gamma Receptor signaling pathway is involved in the internalization of, and proinflammatory output in response to *B*. *burgdorferi*.

### The phosphatase, SHIP1, represses the phagocytosis of *B*. *burgdorferi* but does not affect the induction of TNF

**Fig 7:**
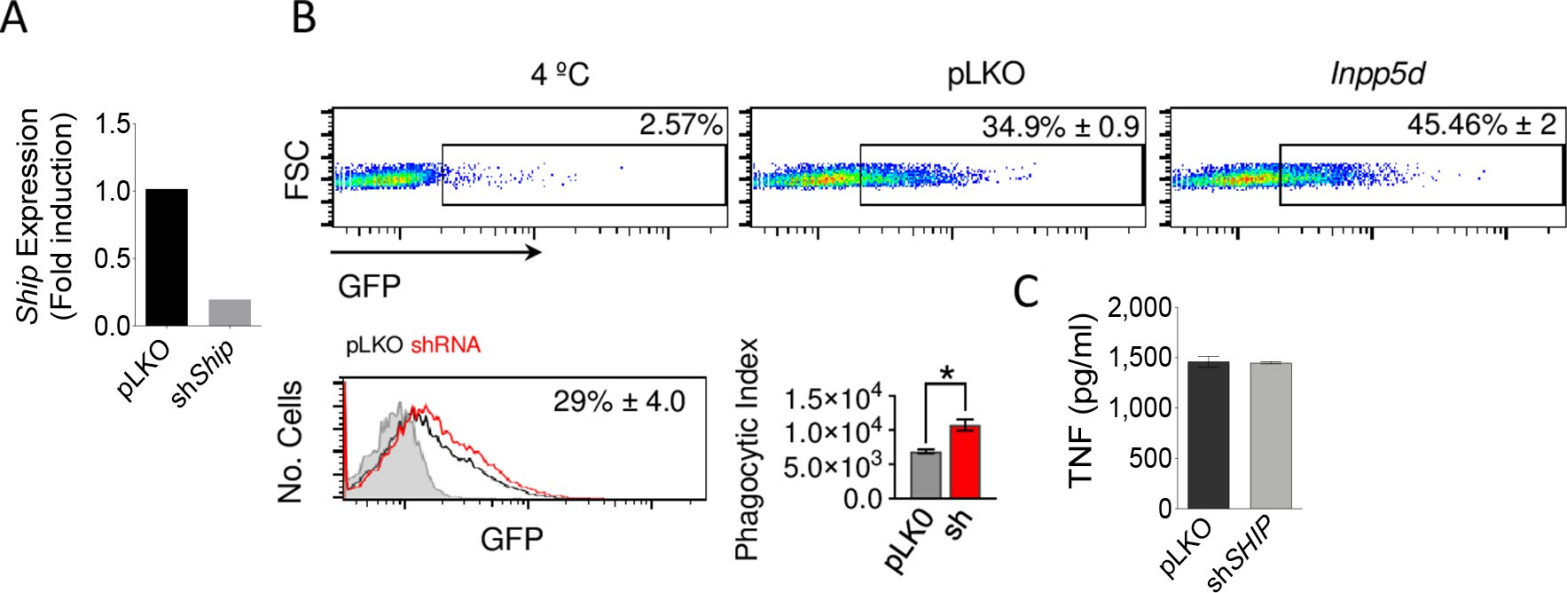
The phosphatase, SHIP1, represses the phagocytosis of *B*. *burgdorferi* but does not affect the induction of TNF. (A) Downregulation of Ship (*Inpp5d*) expression in RAW 264.7 cells infected with a lentivirus containing specific shRNA (grey bar) or an empty vector (pLKO.1, black), as determined by qRT-PCR. (H) Phagocytosis of *B*. *burgdorferi* by sh*Ship* RAW264.7 cells (grey bar) and pLKO.1-infected controls (black bar), analyzed as in Fig 5B. The average ± SE of 3 independent experiments is shown. (I) TNF production upon stimulation with *B*. *burgdorferi* by sh*Ship* RAW264.7 cells (grey bar) or pLKO.1 controls (black bar). The data represent the average ± SE of 3 independent experiments.

The myeloid specific phosphatase, SHIP1, modulates several signaling pathways, including those initiated by Fc gamma receptors [25] and other surface receptors [19]. The stimulation of BMMs with *B*. *burgdorferi* induced the downregulation of *Inpp5d* (encoding SHIP1, Table 1) and the protein is present in the phagosomal enriched fractions of human macrophages (Table 2). To address the potential role of SHIP1 on the response of macrophages to the spirochete, we silenced *Inpp5d* in RAW264.7 cells (Fig 7A). We observed an increased capacity of silenced cells to phagocyte *B*. *burgdorferi* (56%; Fig 7B). However, the induction of TNF was not different between control and *Inpp5d*-silenced cells (Fig 7C), indicating that the phosphatase is specifically involved in the internalization of *B*. *burgdorferi*.

## Discussion

Macrophages are key elements of the immune response; they are not only involved in the clearance of the pathogens, but also act as a bridge between the innate and adaptive arms of the immune response. Macrophages harbor a large number of surface receptors, which confer this cell type a high level of plasticity. Upon engagement of pathogen-recognition receptors (PRRs), macrophages produce an array of cytokine/chemokines that orchestrate the behavior of the downstream components of the inflammatory response. Surface receptors that specifically recognize the pathogen recruit signaling molecules that transduce signals and mediate the internalization and clearance of the pathogen. Multiple receptors may intervene in the phagocytosis of the same pathogen [19, 26]. Thus, the overall signature response to a specific pathogen is dictated by the addition of the signals initially emanating from surface receptors, further modulated by the interaction of intracellular signals generated as a result of the internalization of the pathogen [15].

We have identified a variety of receptors implicated in the response of macrophage to *B*. *burgdorferi*. For this purpose, we first analyzed the proteomic content of phagosome-enriched fractions containing *B*. *burgdorferi*. The analysis of these fractions showed that albeit enriched with phagosomes, they still contained plasma-membrane fragments as the result of the processing of the cells. Therefore, we further complemented this approach with the identification of surface receptors regulated by the stimulation of macrophages with the spirochete [15], an approach previously used by Hu and collaborators that led to the identification of MARCO as a phagocytic receptor for *B*. *burgdorferi* [11]. Our approach also allowed us to identify MARCO and uPAR, another receptor previously involved in the uptake of the spirochete [10], as proteins involved in the response of macrophages to *B*. *burgdorferi*. Furthermore, these results also confirmed the role of CR3/CD14 in the response of phagocytic cells to the microorganism.

Our results show that the repertoire of surface proteins involved in this response is complex and composed of several classes of receptors, including C-lectins, scavenger receptors, siglecs and Fc receptors. They also show that some of the identified receptors contribute to the binding of *B*. *burgdorferi*, while others, including the GPI-anchored proteins analyzed, seem to have an accessory role, possibly through their interaction with CR3 [27] and potentially, other receptors [28]. The importance of the common FcRγ-chain in the control of spirochetal burdens in mice has been previously demonstrated albeit in the presence of antibodies [29]. The phenotype shown in this study was attributed to the importance of an ongoing antibody-mediated clearance during Lyme borreliosis. However, since the FcRγ-chain acts as a functional receptor for other surface proteins, including some that we demonstrate are involved in the recognition of *B*. *burgdorferi*, such as CLEC4N [30], these results may reflect the combined effect exerted by this receptor in both opsonic and non opsonic phagocytosis. In fact, we demonstrate that CD64, the high affinity Fc receptor, directly binds *B*. *burgdorferi* suggesting that Fc receptors mediate both antibody-dependent and independent responses in macrophages. The role played by the opsonin-independent phagocytosis of *B*. *burgdorferi* mediated by Fc receptors and those dependent on the FcRγ-chain may be more relevant at the tissue level, once the spirochetes have disseminated to tissues with poor antibody penetration [31].

**Fig 8:**
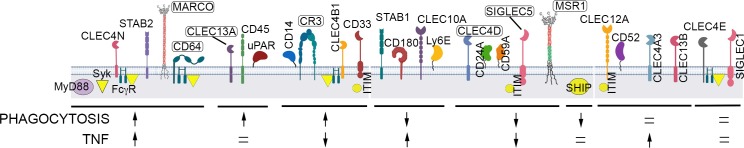
A phagocytic receptor model for B. burgdorferi. Both surface receptors and signaling molecules are represented and grouped according their influence on the internalization of the spirochete and the induction of TNF. The receptors that bind *B*. *burgdorferi* are identified inside a squircle. Those receptors with known signaling partners (i.e. FcRg/Syk) are also identified. The role of the signaling components MyD88, Syk and SHIP are included independently of their associated receptors.

Our approach did not identify an obvious candidate as surface receptor dependent on MyD88, a signaling molecule that accounts for a large portion of the phagocytic activity of macrophages to the spirochete [29, 32]. However, it is possible that some of the receptors herein identified may be dependent on MyD88-derived signals. Further work should defined whether this is the case. In contrast, we further define the Syk-mediated pathway, probably elicited by Fc receptors and certain C-type lectins, as regulating both the internalization of and the induction of proinflammatory cytokines by *B*. *burgdorferi* (Fig 8). The role played by Syk in the internalization of the spirochete confirms previous findings [16]. This signaling pathway is a positive regulator of the proinflammatory activity of macrophages, in contrast to CR3/CD14. This diversity in the role played by different surface receptors on the biosignature of macrophages is further reinforced by the presence of proteins with positive and negative modulatory effects both in the uptake of the spirochete and the ensuing induction of TNF. We previously demonstrated that phagosomes containing *B*. *burgdorferi* are diverse and include CR3 or not [14]. It is feasible that the same would be true and a certain number of different phagosomal compositions would distinctly participate in the induction of proinflammatory cytokines. Overall, both the composition and the regulation of receptors involved in the response of macrophages to *B*. *burgdorferi* provide not only the capacity to eliminate the bacteria, but also the overall inflammatory output of these cells, which could be modulated over time in response to the signals that affect their expression, such as the bacterium itself.

## Methods

### Mice

C57Bl/6 (B6) mice were purchased from Charles River Laboratories (Barcelona, Spain) and bred at CIC bioGUNE. CD64-deficient mice (C.129P2-Fcgr1^tm1Sjv^/Cnrm), in a B6 background [33] were obtained from Dr. J. S. Verbeek and bred at CIC bioGUNE. Euthanasia of mice was performed by CO_2_ inhalation, followed by cardiac exsanguination.

### Bacteria

*B*. *burgdorferi* wild type 297 and Bb914 strains were used for cell stimulations. Bb914 constitutively expresses a GFP cassette stably inserted into cp26 [34]. Bacteria were grown in 5 ml tubes at 34°C in BSK-H medium (Sigma Aldrich, Madrid, Spain) for 3–5 days.

### Cell culture

RAW 264.7 cells (ATCC, Manassas, VA) were maintained in DMEM (Lonza, Barcelona, Spain) supplemented with 10% FCS and 1% penicillin–streptomycin (Thermo Fisher Scientific, Alcobendas, Madrid, Spain). CHO cells stably transfected for human Fcγ Receptor I Alpha (FcgRI, CD64) and transiently transfected for human receptors: MARCO, MSR1, SIGLEC5, CLEC4N, CLEC4D, CLEC4B1, CLEC13A, CLEC10A, Ly6E, CD24a and CD59a were generated using full open reading frames under the CMV promoter (Origene, Herford, Germany). The cells were maintained in Ham’s F-12 medium (Sigma Aldrich) supplemented with 10% FCS, 1% penicillin–streptomycin and 1% L-glutamine. Bone marrow-derived macrophages (BMMs) were generated from 8–12-week-old C57Bl/6 (B6) mice as described [35]. Bone marrow cells were collected from the femoral shafts and incubated in 100 mm x 15 mm Petri dishes (Thermo Fisher Scientific) for 6 days in DMEM supplemented with 10% FCS and 1% penicillin–streptomycin plus 30 ng/ml of M-CSF (Miltenyi Biotec, Bergisch Gladbach, GE). Following incubation, non-adherent cells were eliminated, and adherent macrophages were scraped, counted and seeded in tissue-culture plates. Macrophages were allowed to rest overnight prior to stimulation. Inhibition of Syk kinases was achieved using Piceatannol [36] (30 μM; Sigma Aldrich) one hour prior to the addition of *B*. *burgdorferi*.

Lentiviral particles containing shRNA targeting different receptors (S2 Table; Mission shRNA, Sigma Aldrich) were produced as previously described [37]. Supernatants containing the virus were used to infect RAW 264.7 cells, followed by selection with puromycin at a concentration of 3 μg/ml to generate stable lines. Cell lines infected with the empty vector, pLKO.1, were used as controls. Gene silencing in BMMs was achieved by lentivirus addition at days 3 and 6 of the differentiation process.

The level of expression of the receptor genes was tested either by surface staining or qRT-PCR. Total RNA was purified by Trizol extraction and reverse transcribed with the M-MLV Kit (Thermo Fisher Scientific). Real-time PCRs were performed using perfeCTa SYBR Green SuperMix Low ROX (Quantabio, Beverly, MA) on a QuantStudio 6 real-time PCR System (Thermo Fisher Scientific). The mRNA relative quantification was calculated using the ΔΔCt method. PCR efficiency was always between 90 and 110%. The primers used are listed in S2 Table.

Human monocytes were purified from buffy coats of healthy blood donors by positive selection using a human CD14 purification kit (Miltenyi Biotec), as described [15]. To obtain macrophages, purified human monocytes were incubated for 8 days in RPMI 1640 medium (Lonza) supplemented with 10% FCS, 2.4 mM L-glutamine, 1% penicillin-streptomycin and 30 ng/ml of human M-CSF (Miltenyi Biotec). The cells were rested overnight before stimulation. Blocking of human Fcγ Receptor I Alpha was performed by incubation of human monocyte-derived macrophages (hMAC) with 2 μg/ml of anti-human CD64 (Antibodies.com, Cambridge, UK) one hour prior to the addition of *B*. *burgdorferi*.

### Ethics statement

All work performed with animals was approved by an Órgano Habilitado (Comité de Bioética y Bienestar Animal, CBBA/IACUC, at CIC bioGUNE) and the competent authority (Diputación de Bizkaia) following European (Article 55 of the regulation (EC) no 882/2004 of the European Parliament and of the Council; Article 25 of the regulation (EC) no 1/2005 of the Council; Council Directive 98/81/EC; Council Directive 2003/65/CE) and Spanish (Ley 14/2007, de 3 de julio, de Investigación biomédica; Real Decreto 178/2004, de 30 de enero, sobre utilización confinada de organismos modificados genéticamente; Real Decreto 664/1997, de 12 de mayo, sobre la exposición a agentes biológicos de riesgo durante el trabajo; Real Decreto 363/1995, de 10 de marzo, sobre clasificación de sustancias químicas peligrosas; Real Decreto 53/2013, del 1 de febrero, sobre protección de los animales utilizados para experimentación y otros fines científicos; Ley 32/2007, de 7 de noviembre, sobre el cuidado de los animales en experimentación) directives, under the following protocols: P-CBG-CBBA-1812, P-CBG-CBBA-2112 and P-CBG-CBBA-0917. CIC bioGUNE’s Animal Facility is accredited by AAALAC Intl.

All human samples were obtained after approval by the Basque Country’s Ethics committee following the Helsinki convention. Donors signed an informed consent form and were anonymized to the authors.

### Phagocytosis assays

Phagocytosis assays were performed as previously described [14]. Experiments were performed in DMEM medium without serum or antibiotics. The day before the assay, the cells were seeded at a density of 3 × 10^5^ cells per ml in complete DMEM media. After 16 h, the cell medium was changed to DMEM without supplements and the cells were rested for 1 h. GFP expressing *B*. *burgdorferi* were then added to the cells at a multiplicity of infection (m.o.i.) of 25 and incubated at 4°C for 15 min followed by 37°C for 2 h. The cells were then washed to eliminate surface bacteria and analyzed by flow cytometry in a BD FACS Canto II cytometer (BD Biosciences, San Agustín de Guadalix, Madrid, Spain). The data were analyzed using FlowJo for Mac, version 10.5.3 (FlowJo, Ashland, OR). The phagocytic index was calculated following the formula: %GFP cells (Test) x MFI (Test)—%GFP cells (4°C control) x MFI (4°C control), as described by Hovius and cols. [10].

### Binding assays

3.5 x 10^5^ silenced RAW 264.7 cells were incubated on ice in a rotary shaker with GFP-*B*. *burgdorferi* (m.o.i = 25) in 5 ml tubes (Falcon) in 1 ml of Ham’s F12 medium without serum or antibiotics. After 45 min, the cells were analyzed by flow cytometry. Cell lines infected with the empty vector, pLKO.1 were used as binding controls whereas medium containing GFP-*B*. *burgdorferi* was used as blank.

### Confocal microscopy

Following incubation of CHO cells with *B*. *burgdorferi-*GFP 297 at 37°C, the cells were washed with PBS, fixed with 4% paraformaldehyde for 20 min, permeabilized with PBS containing 0,3% Triton X-100 (VWR, Llinars del Vallés, Barcelona, Spain) and stained with rhodamine phalloidin and DAPI for 10 min at 37°C (Thermo Fisher Scientific) (1:300). After extensive washing in PBS, the cells were mounted using the Prolong Gold Antifade mounting reagent (Thermo Fisher Scientific). The images were obtained employing a Leica TCS SP8 confocal system (Leica Microsystems, Madrid, Spain).

### Phagosome purification

Isolation of *B*. *burgdorferi*-containing phagosomes was performed as previously described [38]. Twenty million BMMs were stimulated with *B*. *burgdorferi-*GFP at an m.o.i. of 50 for 2h. The cells were washed twice with PBS, scraped and centrifuged. The cell pellet was resuspended in 1 ml of homogenization buffer (20mM HEPES, 0.5mM EGTA, 8.5% sucrose plus a protease inhibitor cocktail (Sigma-Aldrich) and lysed by passage through a 27 G needle until at least a 70% of breakage was observed. Cell debris and nuclei were removed by centrifugation at 500 x *g* and treated with DNAse I. The supernatant was layered on top of a discontinuous sucrose gradient (50%, 45%, 40%, 35%, 32%, 15%) and centrifuged at 100,000 x *g* for 1h at 4°C. One ml fractions were collected from the top and centrifuged in 20 ml of homogenization buffer without sucrose at 110,000 x *g* for 1 h at 4°C. Each pellet was then resuspended in 100 μl of homogenization buffer without sucrose and analyzed.

For *B*. *burgdorferi*-containing phagosome purification from hMACs, we followed a protocol from Vinet and Descoteaux [39] with some modifications. The nuclei-free phagosome sample was mixed with an equal volume of 50% sucrose, placed in the sucrose gradient between the 15% and 40% sucrose layers, added an 8.5% sucrose layer, centrifuged at 100,000 x *g* for 1 h and the fractions recovered from the top to the bottom, as before.

### Cryo-electron microscopy

A freshly glow-discharged 300-mesh holey-carbon coated grid (R 2/2 on Copper 300 mesh grids; Quantifoil) was placed inside the chamber of a Vitrobot Mark II (FEI Company, USA), and maintained at 8°C and at a relative humidity close to saturation (85% rH) to prevent drying artefacts in the blotting process. Four microliters of sample solution were absorbed onto the grid for 30 seconds. After incubation, most of the liquid on the grid was removed by blotting (blot time was 3 seconds, number of blots was set to 1, drain time was zero and blot offset was -3 mm) with absorbent standard Vitrobot filter paper (Ø55/20mm, Grade 595, Thermo Fisher Scientific—FEI) to create an “ultra-thin liquid film” (i.e., typically bellow ~500 nm vitreous-ice film thicknesses). The grid was then abruptly plunged into a liquid ethane bath, previously cooled with liquid nitrogen at approximately -170°C. Once the specimen was frozen, the vitrified grids were removed from the plunger and stored under liquid nitrogen inside a cryo-grid storage box.

For imaging under cryo conditions, a vitrified grid was cryo-transferred in a 626 DH cryo transfer holder (Gatan Inc.) and manually analyzed at liquid nitrogen temperature (-174 ˚C) on a JEM-2200FS/CR (JEOL Europe, Croissy-sur-Seine, France) transmission electron microscope. This microscope is equipped with a field emission gun (FEG) operated at 200 kV and an in-column Ω energy filter. During imaging, no-tilted zero-loss two-dimensional (2D) images were recorded under low-dose conditions, utilizing the ‘Minimum Dose System (MDS)’ of Jeol software, with a total dose on the order of 10–20 electrons/Å^2^ per exposure, at defocus values ranging from 1.5 to 4.0 μm. The in-column Omega energy filter of the microscope allowed to record images with improved signal-to-noise ratio (SNR) by zero-loss filtering, using an energy selecting slit width of 30 eV centered at the zero-loss peak of the energy spectra. Digital images were recorded on a 4K × 4K (15 μ*m* pixels) Ultrascan4000 charge-coupled device (CCD) camera (Gatan Inc.) using DigitalMicrograph (Gatan Inc.) software, at different nominal magnifications from 4000× to 60,000×.

### Immunoblotting

Ten μl of each gradient fraction was loaded onto a 4–20% Bis-Tris protein gel (Thermo Fisher Scientific). The separated proteins were then transferred to nitrocellulose membranes and blocked with TBST-5% milk for 1h. The membranes were then incubated with the primary antibody followed by HRP-labeled secondary antibodies, and developed with ECL substrate (Bio-Rad, Alcobendas, Madrid). The following antibodies were used: Anti-human CD64 (Antibodies.com; 1:2,000), anti-GFP (Roche, Madrid, Spain; 1:1,000), anti-human CD11b (clone 23F-12; Santa Cruz Biotechnology, Heidelberg, Germany; 1:200), anti-mouse CD11b (clone M-19; Santa Cruz Biotechnology; 1:1,000), anti-human LAMP1 (clone H4A3; Developmental Studies Hybridoma Bank, University of Iowa, Iowa City, IA; 1:500), anti-mouse LAMP2 (clone GL2A7; Abcam, Cambridge, UK; 1:1,000), anti-mouse FcγR (Merck, Tres Cantos, Madrid, Spain; 1:1,000), anti-human and mouse MyD88 (clone HFL-296; Santa Cruz Biotechnology; 1:1,000), anti-mouse CD14 (clone M-305; Santa Cruz Biotechnology; 1:1,000).

The presence of rabbit IgG on the GFP-*B*. *burgdorferi* preparations used for phagocytosis was tested using an anti-rabbit IgG conjugated to alkaline phosphatase (Cell Signaling Technology; 1/1,000) and normal rabbit IgG as positive control (Cell Signaling Technology).

### Proteomic analysis

#### In solution digestion

Phagosome-enriched proteins were extracted using 7M urea, 2M thiourea and 4% CHAPS. Samples were incubated for 30 min at RT under agitation and digested following the filter-aided FASP protocol described by Wisniewski et al [40] with minor modifications. Trypsin was added to a trypsin:protein ratio of 1:10, and the mixture was incubated overnight at 37°C, dried in an RVC2 25 speedvac concentrator (Christ, Osterode am Harz, Germany), and resuspended in 0.1% Formic Acid.

#### Mass spectrometry analysis

The samples were submitted to LC-MS label-free analysis using two different platforms. First, peptide separation was performed on a nanoACQUITY UPLC System (Waters, Cerdanyola del Vallés, Barcelona, Spain) connected on-line to an LTQ Orbitrap XL mass spectrometer (Thermo Fisher Scientific) using the same parameters described before [15]. In addition, the samples were analyzed in a hybrid trapped ion mobility spectrometry–quadrupole time of flight mass spectrometer (timsTOF Pro with PASEF, Bruker, Rivas Vaciamadrid, Madrid, Spain) coupled online to a nanoElute liquid chromatograph (Bruker). The samples (200 ng) were directly loaded in a 25 cm, 75 μm ID Odyssey C18 nano column with an integrated emitter (IonOpticks, Parkville, Victoria, Australia) and resolved at 400 nl/min with a 90 min gradient. The column was heated to 50°C using an oven.

Searches were carried out using the Mascot search engine (Matrix Science, London, UK) through Proteome Discoverer software 1.4 (Thermo Fisher Scientific). Orbitrap RAW files were directly loaded into the program, whereas mgf files generated by DataAnalysis software (Bruker) were used for timsTOF searches. Orbi searches were carried out with precursor and fragment tolerances of 10 ppm and 0.5 Da, whereas 50 ppm and 0.05 Da were used for timsTOF runs. A database consisting of human (Uniprot/Swissprot) and *Borrelia burgdorferi* (Uniprot/Swissprot and Uniprot/TrEMBL) entries (2018_10 release) was used for the searches. Only proteins identified with at least one peptide with FDR < 1% were considered. Spectral counts for each protein (the number of identified spectra matching to peptides from that protein, also named SpC or PSMs) were normalized against protein length. Then, these values were converted to Normalized Spectral Abundance Factor (NSAF) values by normalizing them against the sum of normalized spectral counts in each of the runs.

### TNF ELISA

The levels of TNF produced by *B*. *burgdorferi* stimulation were determined by capture ELISA using the DuoSet II kit (R&D Systems, Minneapolis, MN) according to the manufacturer’s recommendations.

### Statistical analysis

The results are presented as the means ± SE (standard error). Significant differences between means were calculated with the Student’s t test. Multiple comparisons were analyzed by ANOVA, followed by pairwise comparisons. A p value < 0.05 was considered significant. All statistical calculations were performed with GraphPad Prism ver. 8.

## Supporting information

S1 Fig Downregulation of receptor expression in silenced RAW 264.7 cells. (A) Downregulation of surface receptors upon lentiviral infection containing specific sh*RNA* (grey bar) compared to lentivirus containing the empty vector (pLKO.1, black), as determined by qRT-PCR. (B) Surface expression (MFI) of MSR1, SIGLEC1 and uPAR in sh*RNA*- (grey bar) and pLKO.1 (black bar)-infected RAW 264.7 cells.
(TIF)

S2 Fig Phagocytosis of *B*. *burgdorferi* by C-type Lectin-silenced RAW 264.7 cells. (A) Percentage of GFP-positive RAW 264.7 cells in sh*RNA*- (right panel) or pLKO.1-infected (middle panel) cells. The 4°C control is represented on the left panels. The numbers represent the average ± SE of 3 determinations. (B) Histograms representing phagocytosis by sh*RNA*- (red histogram) and control-infected (black histograms) RAW 264.7 cells. The gray histogram represents the 4°C control. The numbers represent the average reduction in MFI ± SE of 3 determinations. (C) Phagocytic index of sh*RNA*- (grey bars) and pLKO.1-infected (red bars) RAW 264.7 cells. The data represent the average ± SE of 3 determinations.
(TIF)

S3 Fig Phagocytosis of *B*. *burgdorferi* by Scavenger receptors-silenced RAW 264.7 cells. (A) Percentage of GFP-positive RAW 264.7 cells in sh*RNA*- (right panel) or pLKO.1-infected (middle panel) cells. The 4°C control is represented on the left panels. The numbers represent the average ± SE of 3 determinations. (B) Histograms representing phagocytosis by sh*RNA*- (red histogram) and control-infected (black histograms) RAW 264.7 cells. The gray histogram represents the 4°C control. The numbers represent the average reduction in MFI ± SE of 3 determinations. (C) Phagocytic index of sh*RNA*- (grey bars) and pLKO.1-infected (red bars) RAW 264.7 cells. The data represent the average ± SE of 3 determinations.
(TIF)

S4 Fig Phagocytosis of *B*. *burgdorferi* by Siglec-silenced RAW 264.7 cells. (A) Percentage of GFP-positive RAW 264.7 cells in sh*RNA*- (right panel) or pLKO.1-infected (middle panel) cells. The 4°C control is represented on the left panels. The numbers represent the average ± SE of 3 determinations. (B) Histograms representing phagocytosis by sh*RNA*- (red histogram) and control-infected (black histograms) RAW 264.7 cells. The gray histogram represents the 4°C control. The numbers represent the average reduction in MFI ± SE of 3 determinations. (C) Phagocytic index of sh*RNA*- (grey bars) and pLKO.1-infected (red bars) RAW 264.7 cells. The data represent the average ± SE of 3 determinations.
(TIF)

S5 Fig Phagocytosis of *B*. *burgdorferi* by GPI-anchored proteins-silenced RAW 264.7 cells. (A) Percentage of GFP-positive RAW 264.7 cells in sh*RNA*- (right panel) or pLKO.1-infected (middle panel) cells. The 4°C control is represented on the left panels. The numbers represent the average ± SE of 3 determinations. (B) Histograms representing phagocytosis by sh*RNA*- (red histogram) and control-infected (black histograms) RAW 264.7 cells. The gray histogram represents the 4°C control. The numbers represent the average reduction in MFI ± SE of 3 determinations. (C) Phagocytic index of sh*RNA*- (grey bars) and pLKO.1-infected (red bars) RAW 264.7 cells. The data represent the average ± SE of 3 determinations.
(TIF)

S6 Fig Flow cytometry analysis of CD11b expression in sh*Clec10a*, sh*Stab1*, sh*Ly6e*, sh*Clec4b1* and sh*Cd33* RAW 264.7 cells. Sh*RNA* infected RAW 264.7 cells (red histogram) and pLKO.1-infected controls (black histogram) were analyzed for CD11b expression by flow cytometry. The gray histogram represents the unstained control. The data represent at least 3 independent determinations.
(TIF)

S1 Table shRNAs used. (DOCX)

S2 Table Primers used. All primers were used at an annealing temperature of 60°C, except for *Stab1* and *Plaur* (58°C).
(DOCX)
