# Supplementary figures and images for: Regulation of macrophage activity by surface receptors contained within Borrelia burgdorferi-enriched phagosomal fractions
Source: PLoS Pathog. 2019 Nov 18;15(11):e1008163. doi: 10.1371/journal.ppat.1008163 (PMC6886865; doi:10.1371/journal.ppat.1008163)

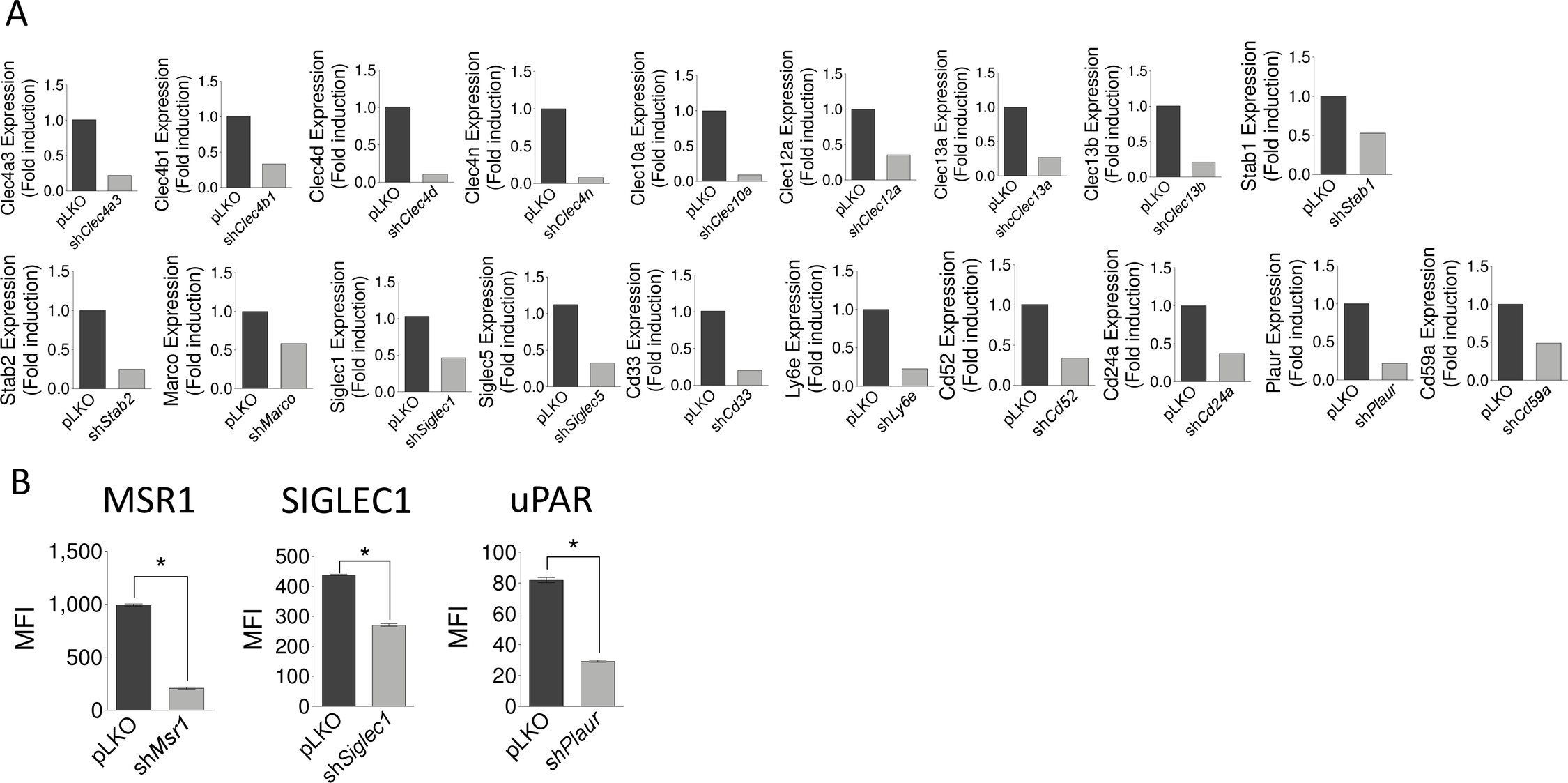

Supplement: S1 Fig — (A) Downregulation of surface receptors upon lentiviral infection containing specific shRNA (grey bar) compared to lentivirus containing the empty vector (pLKO.1, black), as determined by qRT-PCR. (B) Surface expression (MFI) of MSR1, SIGLEC1 and uPAR in shRNA- (grey bar) and pLKO.1 (black bar)-infected RAW 264.7 cells. (TIF) [file ppat.1008163.s001.tif]

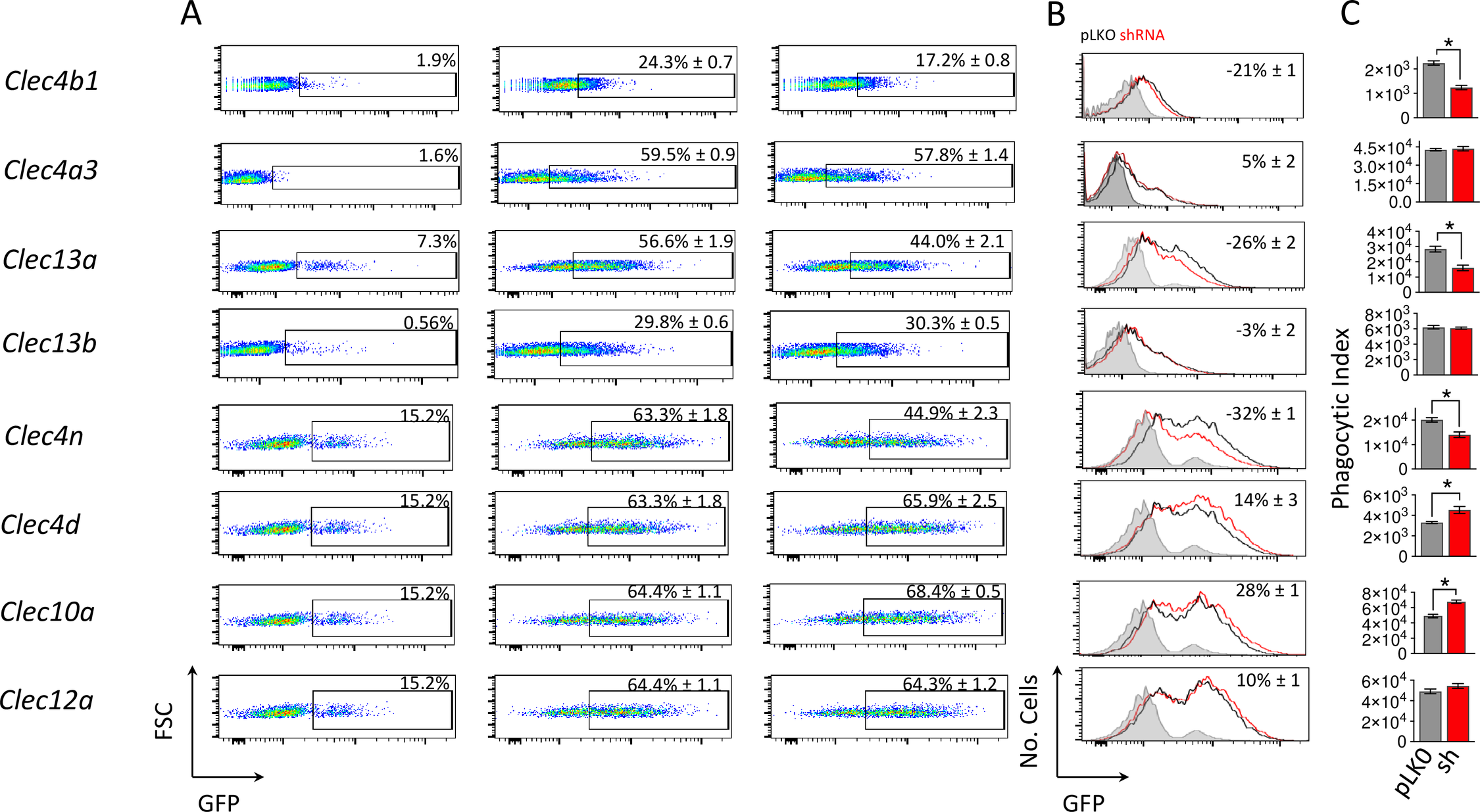

Supplement: S2 Fig — (A) Percentage of GFP-positive RAW 264.7 cells in shRNA- (right panel) or pLKO.1-infected (middle panel) cells. The 4°C control is represented on the left panels. The numbers represent the average ± SE of 3 determinations. (B) Histograms representing phagocytosis by shRNA- (red histogram) and control-infected (black histograms) RAW 264.7 cells. The gray histogram represents the 4°C control. The numbers represent the average reduction in MFI ± SE of 3 determinations. (C) Phagocytic index of shRNA- (grey bars) and pLKO.1-infected (red bars) RAW 264.7 cells. The data represent the average ± SE of 3 determinations. (TIF) [file ppat.1008163.s002.tif]

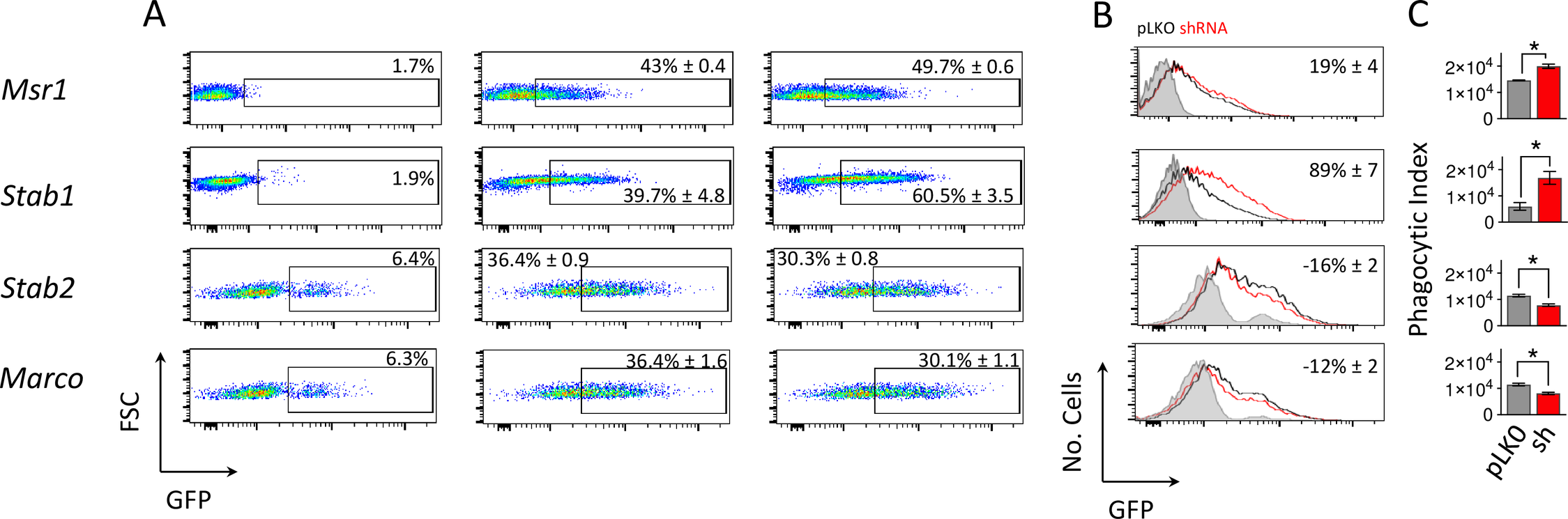

Supplement: S3 Fig — (A) Percentage of GFP-positive RAW 264.7 cells in shRNA- (right panel) or pLKO.1-infected (middle panel) cells. The 4°C control is represented on the left panels. The numbers represent the average ± SE of 3 determinations. (B) Histograms representing phagocytosis by shRNA- (red histogram) and control-infected (black histograms) RAW 264.7 cells. The gray histogram represents the 4°C control. The numbers represent the average reduction in MFI ± SE of 3 determinations. (C) Phagocytic index of shRNA- (grey bars) and pLKO.1-infected (red bars) RAW 264.7 cells. The data represent the average ± SE of 3 determinations. (TIF) [file ppat.1008163.s003.tif]

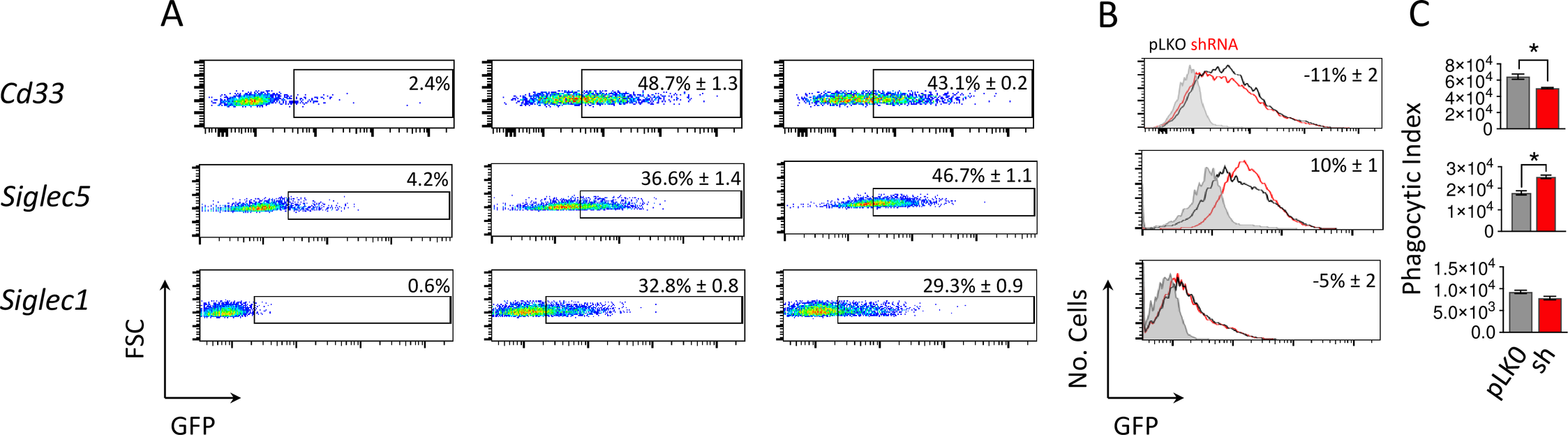

Supplement: S4 Fig — (A) Percentage of GFP-positive RAW 264.7 cells in shRNA- (right panel) or pLKO.1-infected (middle panel) cells. The 4°C control is represented on the left panels. The numbers represent the average ± SE of 3 determinations. (B) Histograms representing phagocytosis by shRNA- (red histogram) and control-infected (black histograms) RAW 264.7 cells. The gray histogram represents the 4°C control. The numbers represent the average reduction in MFI ± SE of 3 determinations. (C) Phagocytic index of shRNA- (grey bars) and pLKO.1-infected (red bars) RAW 264.7 cells. The data represent the average ± SE of 3 determinations. (TIF) [file ppat.1008163.s004.tif]

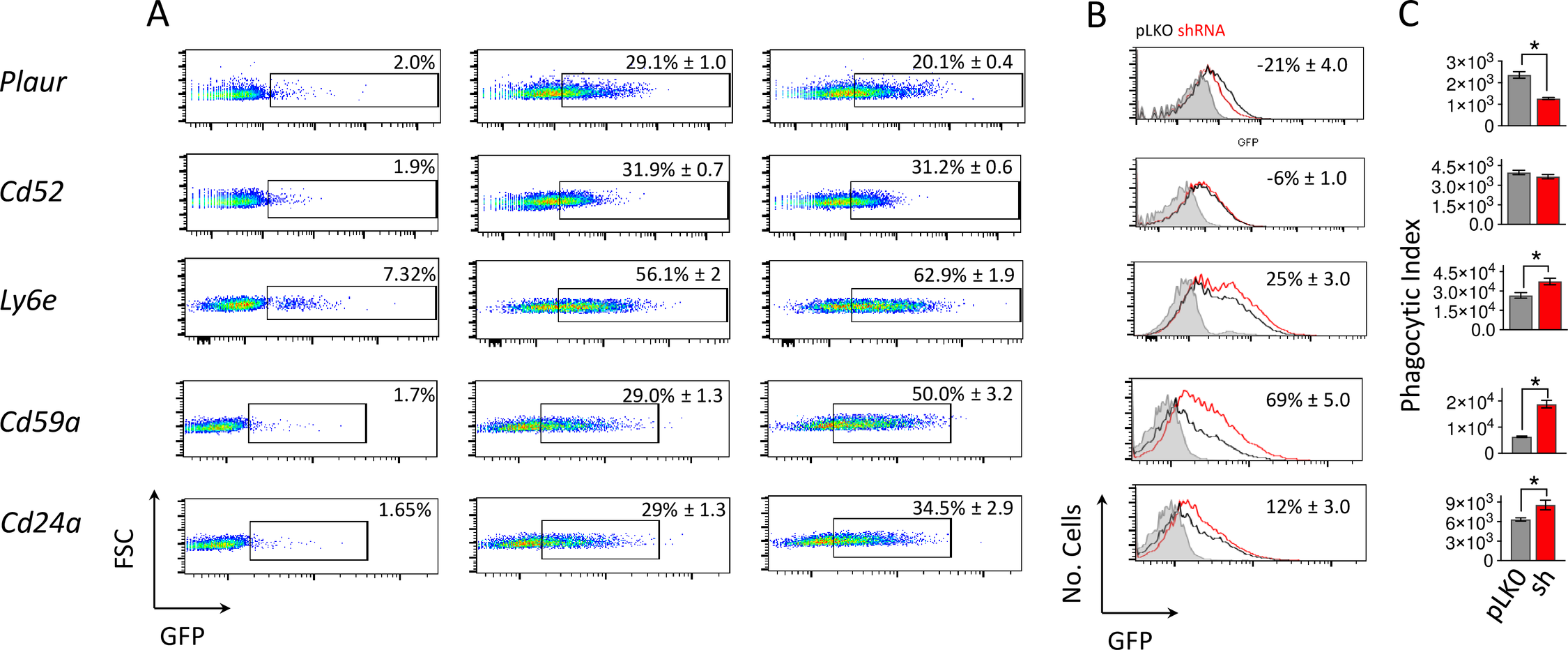

Supplement: S5 Fig — (A) Percentage of GFP-positive RAW 264.7 cells in shRNA- (right panel) or pLKO.1-infected (middle panel) cells. The 4°C control is represented on the left panels. The numbers represent the average ± SE of 3 determinations. (B) Histograms representing phagocytosis by shRNA- (red histogram) and control-infected (black histograms) RAW 264.7 cells. The gray histogram represents the 4°C control. The numbers represent the average reduction in MFI ± SE of 3 determinations. (C) Phagocytic index of shRNA- (grey bars) and pLKO.1-infected (red bars) RAW 264.7 cells. The data represent the average ± SE of 3 determinations. (TIF) [file ppat.1008163.s005.tif]

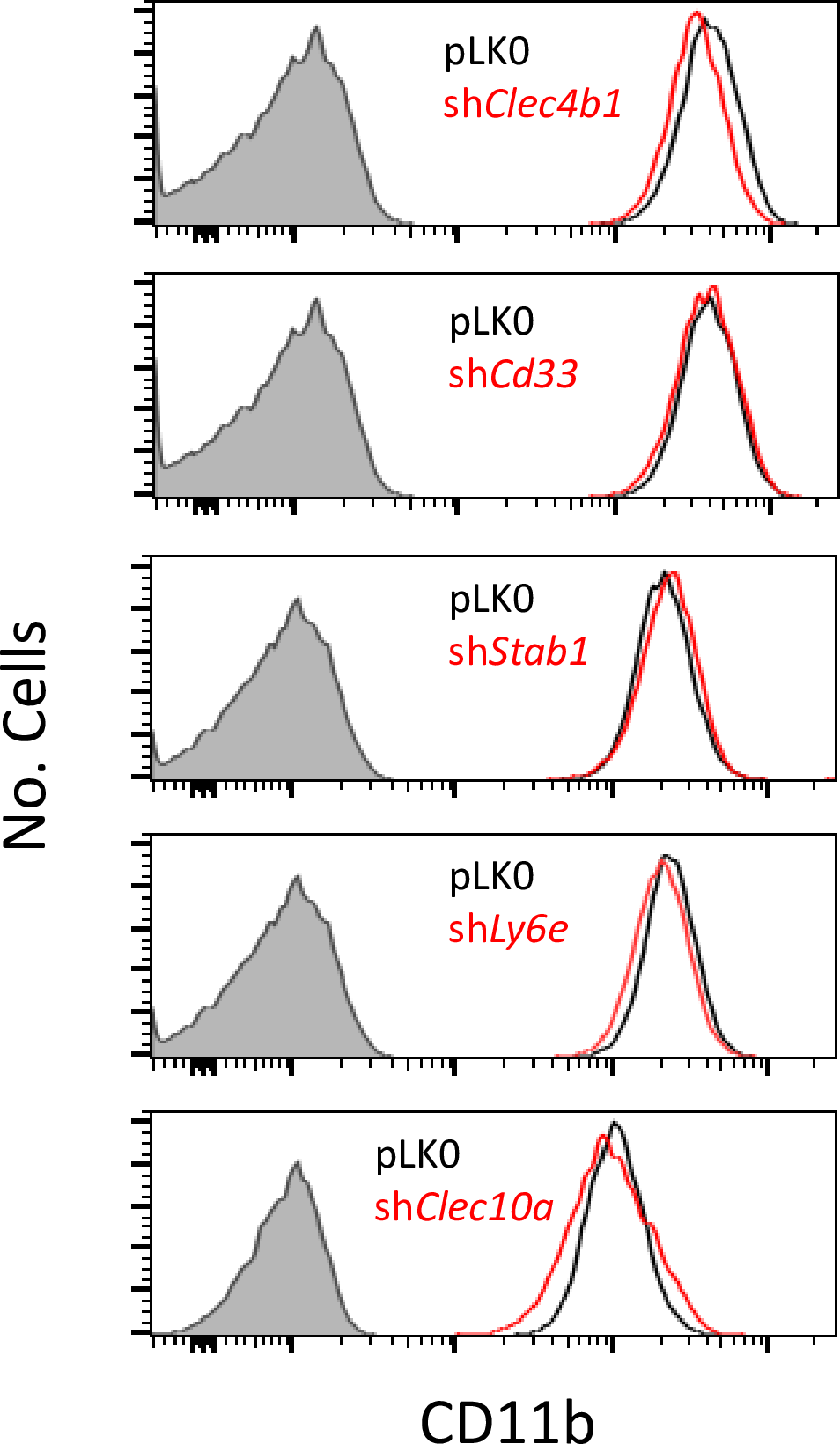

Supplement: S6 Fig — ShRNA infected RAW 264.7 cells (red histogram) and pLKO.1-infected controls (black histogram) were analyzed for CD11b expression by flow cytometry. The gray histogram represents the unstained control. The data represent at least 3 independent determinations. (TIF) [file ppat.1008163.s006.tif]
