# Supplementary material for: Regulation of macrophage activity by surface receptors contained within Borrelia burgdorferi-enriched phagosomal fractions
Source: PLoS Pathog. 2019 Nov 18;15(11):e1008163. doi: 10.1371/journal.ppat.1008163 (PMC6886865; doi:10.1371/journal.ppat.1008163)
Supplement: S1 Table — (DOCX) [file ppat.1008163.s007.docx]

**S1 Table**. sh*RNA*s used.

| Gene | Gene Bank Accession Nº | Mission shRNA catalogue Nº |
| --- | --- | --- |
| *Fcer1g* | NM_010185.4 | TRCN0000067588 |
| *Fcgr1* | NM_010186.5 | TRCN0000067678 |
| *Ptprc* | NM_011210.4 | TRCN0000029929 |
| *Inpp5d* | NM_010566.3 | TRCN0000436483 |
| *Clec4a3* | NM_153197.2 | TRCN0000109845 |
| *Clec4b1* | NM_027218.2 | TRCN0000436087 |
| *Clec4d* | NM_010819.4 | TRCN0000304453 |
| *Clec4n* | NM_020001.2 | TRCN0000066787 |
| *Clec10a* | NM_010796.3 | TRCN0000067028 |
| *Clec12a* | NM_177686.4 | TRCN0000249250 |
| *Cd302* | NM_025422.4 | TRCN0000176008 |
| *Ly75* | NM_013825.3 | TRCN0000375342 |
| *Stab1* | NM_138672.2 | TRCN0000215721 |
| *Stab2* | NM_138673.3 | TRCN0000109510 |
| *Marco* | NM_010766.3 | TRCN0000424243 |
| *Msr1* | NM_031195.2 | TRCN0000431188 |
| *Siglec5* | NM_145581.2 | TRCN0000094304 |
| *Cd33* | NM_021293.1 | TRCN0000094368 |
| *Siglec1* | NM_011426.1 | TRCN0000094864 |
| *Cd52* | NM_013706.2 | TRCN0000077003 |
| *Ly6e* | NM_008529.2 | TRCN0000101140 |
| *Cd59a* | NM_007652.5 | TRCN0000329358 |
| *Cd24a* | NM_009846.2 | TRCN0000332785 |
| *Plaur* | NM_011113.3 | TRCN0000362760 |
