# Supplementary material for: Regulation of macrophage activity by surface receptors contained within Borrelia burgdorferi-enriched phagosomal fractions
Source: PLoS Pathog. 2019 Nov 18;15(11):e1008163. doi: 10.1371/journal.ppat.1008163 (PMC6886865; doi:10.1371/journal.ppat.1008163)
Supplement: S2 Table — All primers were used at an annealing temperature of 60°C, except for Stab1 and Plaur (58°C). (DOCX) [file ppat.1008163.s008.docx]

**S2 Table**. **Primers used.** All primers were used at an annealing temperature of 60 ºC, except for *Stab1* and *Plaur* (58 ºC).

| Gene | Primers |
| --- | --- |
| *Ptprc* | 5’-GATCCACTGCTGGGCTTCA-3’  5’-GAACATGCTGCCAATGGTTCT-3’ |
| *Inpp5d* | 5’-CCAGGGCAAGATGAGGGAGA-3’  5’-GGACCTCGGTTGGCAATGTA-3’ |
| *Clec4a3* | 5’-GGGCAACAGCTCCAGACTT-3’  5’-TCTTATTCTCCGGTGCTCTGA-3’ |
| *Clec4b1* | 5’-GGAACCAAGTAGCAGTGGGA-3’  5’-CCTCAGCACCTGTTTCATTG-3’ |
| *Clec4d* | 5’-GGAAAGTCATTCCAGACCCA-3’  5’-AAGACGCCATTTAACCCACA-3’ |
| *Clec4n* | 5’-AGAAGATTTACCTATGAGTGCCTGT-3’  5’-GGCTAGGAAAAGAACGGCCT-3’ |
| *Clec10a* | 5’-AACCTTCCGCTGGATCTGTG-3’  5’-GTTGAGACCGGGTAGGAGGA-3’ |
| *Clec12a* | 5’-GCAAAGGGCCAAGGAAGAAC-3’  5’-TATACAGCTCTCGGCACAGC-3’ |
| *Cd302* | 5’-ATCCAGGATGGTTCCTGTTCTG-3’  5’-ACTTGCCACAAAAACTGAGCC-3’ |
| *Ly75* | 5’-GATAAGGCTGGGCACAAAGG-3’  5’-CTGTGACTCCACCGTCATGC-3’ |
| *Stab1* | 5’- CCACTCCAAATGAAGACTTG -3’  5’- CTACTCATGTGGTTACGATTC -3’ |
| *Stab2* | 5’-GCTGCAAGTCCTCATGTCCT-3’  5’-TTCTGTGGCACAAACAGGGT-3’ |
| *Marco* | 5’-TTAGCAGCTATGGAGGTGGC-3’  5’-GACACACTGATGACCTCTCGG-3’ |
| *Msr1* | 5’-ACCTCCTGTTGCTTTGCTGT-3’  5’-ACACGGAACGCTTCCAGAAT-3’ |
| *Siglec5* | 5’-TGTGCGTCTTTGTAGCCTGC-3’  5’-CACTGGAGAGCCGCTGAAT-3’ |
| *Cd33* | 5’-CAGAGCCCAAGAATCAGGAG-3’  5’-CCTTCTCTGGAGCAGGTGTC-3’ |
| *Siglec1* | 5’-GTCTCCAGGAAGGTGGTCAG-3’  5’-CAGGGCTGATACTGGCTTCT-3’ |
| *Cd52* | 5’-GGTGGAGGTGCTGTTTTTGT-3’  5’-GCCCAGGAAGATTTCAGGAT-3’ |
| *Ly6e* | 5’-TCAGGGAATGAACTTGCTCC-3’  5’-TCGGTATTATCTTCGGGGC-3’ |
| *Cd59a* | 5’-GGAATGCAAGTGTATCAAAGGTGT-3’  5’-CCCCAAGGATCCGTCACTTT-3’ |
| *Cd24a* | 5’-CCCCAAATGGCAACCACAAG-3’  5’-TCAAACCTTTCACGCGTCCT-3’ |
| *Plaur* | 5’- TCTGGATCTTCAGAGCTTTC -3’  5’- GCCTCTTACGGTATAACTCC -3’ |
